# A systematic review of Chinese randomized clinical trials of SSRI treatment of depression

**DOI:** 10.1186/s12888-014-0245-4

**Published:** 2014-08-27

**Authors:** Ying Zhang, Thomas Becker, Yongchun Ma, Markus Koesters

**Affiliations:** Department of Psychiatry II, Ulm University, Ludwig-Heilmeyer-Str.2, 89312 Guenzburg, Germany; Tongde Hospital of Zhejiang Province, 234 Gucui Road, 310012 Hangzhou, PR China

**Keywords:** Systematic review, Risk of bias assessment, China, Antidepressants

## Abstract

**Background:**

Selective serotonin reuptake inhibitors (SSRIs) have become the most frequently used antidepressants in China in recent decades. This systematic review and meta-analysis examined the efficacy and tolerability of SSRIs in Chinese studies and the quality of Chinese randomized controlled trials.

**Methods:**

Major Western and Chinese electronic databases were searched for double-blind, parallel group randomised controlled trials (RCTs) comparing SSRIs (fluoxetine, citalopram, escitalopram, fluvoxamine, paroxetine, or sertraline) with other antidepressants such as SSRI, Selective Noradrenaline Reuptake Inhibitor (SNRI), tricyclic antidepressant (TCA), Traditional Chinese Medicine (TCM) and/or placebo. Response, remission, and dropout rates due to side effects were defined as primary outcomes. Mean total Hamilton Rating Scale of Depression (HAMD) scores at endpoint, overall dropout rates and total Treatment Emergent Symptom Scale (TESS) scores were defined as secondary outcomes. Data were combined with random effects models. Risk of bias was assessed by the Cochrane evaluation tool. Quality of reports was assessed by the fulfilment of Consolidated Standards of Reporting Trial (CONSORT) items.

**Results:**

A total of 71 studies were included. Only one study was listed in both Chinese and Western databases. SSRIs were found to be more effective than TCAs. No significant differences were observed regarding dropout rates due to side effects. Using the Cochrane risk of bias tool, adequate methods of sequence generation were described in 16 (23%) studies. All authors failed to report trial registration. Informed consent, sources of funding, email address, protocol, and limitations were also not mentioned in most studies. However, reporting quality improved steadily between 1996 and 2013.

**Conclusions:**

In light of the low trial quality, the findings of a significant advantage of SSRI over TCA in terms of response rate and remission rate should be replicated by large high-quality Chinese studies.

**Electronic supplementary material:**

The online version of this article (doi:10.1186/s12888-014-0245-4) contains supplementary material, which is available to authorized users.

## Background

In recent decades, selective serotonin reuptake inhibitors (SSRIs) have become the first-line antidepressant drug treatment of depression and replaced tricyclic antidepressants (TCA) and monoamine oxidase inhibitors (MAOI) due to fewer side-effects and ease of use in Western countries [1,2]. Also, SSRIs have become the dominant subcategory of antidepressants in China [3–5].

Although most Chinese studies are reported to be underpowered and of low reporting quality [6–10], many Chinese double-blind randomised controlled trials (RCTs) were carried out to examine the effectiveness and safety of SSRIs. Only 7% of published Chinese studies of efficacy and tolerability of antidepressants were included in western meta-analyses. Less than 6% of the Chinese biomedical journals are indexed in MEDLINE [11].

The present systematic review aims to systematically examine the quality of Chinese double-blind RCTs evaluating SSRIs, to examine the efficacy and tolerability of SSRIs compared with other antidepressant agents, including other SSRIs, Traditional Chinese Medicine (TCM), and/or placebo in Chinese populations, and to formulate recommendations for future research.

## Methods

### Search strategy

Chinese Scientific Journals Full-text Database (VIP) and the China National Knowledge Infrastructure (CNKI) were searched using English and Chinese search terms for depression combined with substance and trade names for SSRIs (fluoxetine, citalopram, escitalopram, fluvoxamine, paroxetine, or sertraline). Furthermore, Western databases MEDLINE and EMBASE were searched using the terms “depression” combined with “China” or “Taiwan” (see Table 1). There were no restrictions on language, publication type or publication date. In addition, the Chinese Clinical Trial Registry was searched, and reference lists of studies included were hand searched. Literature search was last updated in May 2013.

**Table 1:** **Search strategies**

**Databases**	**Search terms**
CNKI/VIP	抑郁/depression + fluoxetine/氟西汀/Prozac/百忧解/奥麦伦/奥贝汀/优克/citalopram/西酞普兰/Cipramil/喜普妙/多弗/escitalopram/艾司西酞普兰/Lexapro/来士普/fluvoxamine/氟伏沙明/氟伏草胺/Luvox/兰释/ paroxetine/帕罗西汀/Paxil/Seroxat/赛乐特/sertraline/舍曲林/Zoloft/左洛复/郁乐复
MEDLINE/EMBASE	Depression + China/Taiwan

### Types of studies, interventions and participants

Inclusion criteria: Double-blind, parallel group RCTs comparing SSRIs (fluoxetine, citalopram, escitalopram, fluvoxamine, paroxetine, or sertraline) with other antidepressants such as Selective Noradrenaline Reuptake Inhibitor (SNRI) or TCA etc., TCM (acupuncture, Chinese herbs) and/or placebo as monotherapy were included. Head to head trials of SSRIs were also included. Study participants had to be Chinese adult patients with a primary diagnosis of depression according to DSM, ICD and/or the Chinese Classification of Mental Disorder (CCMD).

Exclusion criteria: In line with the treatment guideline for depression of the National Institute for Health and Clinical Excellence (NICE), studies were excluded if more than 20% of the participants had a primary diagnosis of dysthymia or if more than 15% had a primary diagnosis of bipolar disorder [12]. Trials were excluded if proportions of bipolar or dysthymia patients remained unclear.

### Ethics statement

Given that the current study utilized secondary data reported on the aggregate level, which is readily available in the literature, it was not necessary to obtain research ethics approval.

### Outcome measures and data analysis

In a first step, titles and abstracts were screened by one author (YZ). All articles rated as “potentially relevant” were then retrieved to establish whether they met inclusion criteria. Study inclusion was independently verified by a second rater (YCM). In case of disagreement the final rating was consented by discussion with a third author.

Data concerning participant characteristics, intervention details and outcome measures of interest were extracted using a pre-designed form. Data were entered into Microsoft excel and subsequently into Comprehensive Meta Analysis (CMA) 2 [13]. In case of missing data, an attempt was made to contact trial authors in order to obtain further information.

Chinese studies usually report efficacy according to four levels defined by the Chinese Medical Association: “remission” as a HAMD reduction of more than 75%, “significant progress” as a HAMD reduction of 50-74%, “progress” as HAMD reduction of 25-49%, and “ineffective” as HAMD reduction of less than 25%. For the present analysis, remission was defined a priori as a HAMD reduction of more than 75% or, if reported, final rating of HAMD ≤ either 7 or 8 [6]. Response was defined as a HAMD reduction of at least 50%.

Response, remission, and dropout rates due to side effects were defined as primary outcomes for efficacy and tolerability. Mean total HAMD scores at endpoint, overall dropout rates, and total TESS score were defined as secondary outcomes. Hedges’ g was calculated as effect size for continuous data; Mantel-Haenszel Risk ratios (MH RR) and 95% confidence interval (95% CI) were used for dichotomous data. Data were combined in random effects models. Subgroup analyses were planned for different control groups (SSRI, SNRI, TCA, TCM etc.) and for geriatric vs. non-geriatric patients. For the present analysis, the definition of geriatric patients was adopted from the studies included, with some studies including patients of 55 years or older (cf. Additional file 1: Appendix 3). Heterogeneity of treatment effect between studies was investigated using the I^2^ parameter [14] and by visual inspection of the forest plots. I^2^ values of more than 50% were considered to indicate heterogeneity [15].

Risk of bias in the studies included was assessed using the Cochrane Collaboration evaluation tool [16]. Additionally, the fulfilment of Consolidated Standards of Reporting Trials (CONSORT) Items [17] was used to assess reporting quality. Other criteria which are not explicitly mentioned in CONSORT, e.g. obtaining informed consent from participants and contact details for the corresponding author, were also assessed. A meta-regression analysis was carried out to investigate whether the quality of reporting of primary studies as measured with the mean percentage of fulfilled CONSORT items had improved over time. Publication bias was assessed with funnel plots, Beggs rank correlation test, and regression tests.

## Results and discussion

### Results

#### Description of studies

**Figure 1:**
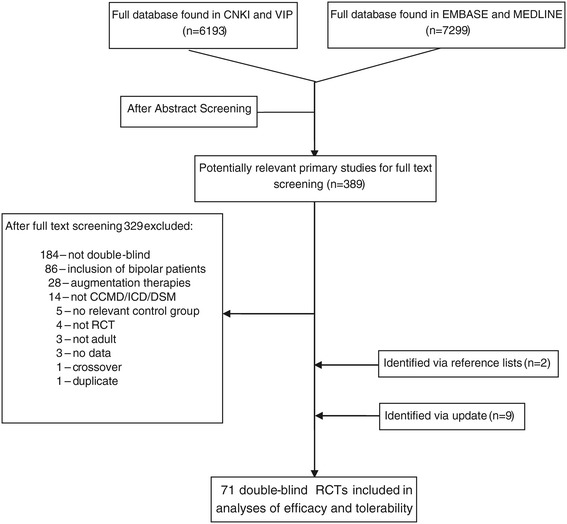
**Flowchart.** RCT: Randomised controlled trials. CCMD: Chinese classification of mental disorders. ICD: International classification of diseases. DSM: Diagnostic and statistical manual of mental disorders. TCM: Traditional Chinese medicine. CNKI: China national knowledge infrastructure. VIP: Chinese scientific journals full-text database.

A total of 13492 citations were identified by the systematic literature search in VIP, CNKI, Chinese Clinical Trail Registry, EMBASE and MEDLINE databases. Of these, 542 remained after abstract screening and were assessed in full text. 71 relevant double-blind RCTs were included. None of the studies listed in the Chinese Clinical Trial Registry was relevant. Only one study was listed in both Chinese and Western databases. Figure 1 illustrates the study selection (a list of all studies included and excluded and naming the reasons for exclusion see Additional file 1: Appendix 1 and 2). If there are multiple reasons for studies to be excluded, only one reason for exclusion was recorded. An overview of included studies is given in Table 2, short descriptions of each study are included in Additional file 1: Appendix 3.

**Table 2:** **Overview of the 71 studies included**

**Characteristics**		
**Median year of publication (Range)**		**2007 (1996–2013)**
**Average duration (Range)**		**6.2 (2–8) weeks**
**Setting**		**Number of studies**
	Inpatients	21
	Outpatients	11
	In- and outpatients	29
	Not reported	10
**Sample**		
	PSD	12
	Only elderly patients (≥55 years)	10
**Study design**		
	Three-arm	6
	Placebo controlled	3
	Double-dummy	17

PSD: Post-stroke depression.

All studies were conducted by authors based in Chinese hospitals. Most of the studies collected data at one hospital, whilst 20 studies reported multisite data. Four studies were published in English, all of them were listed in MEDLINE and EMBASE, and only one of these studies could also be identified via CNKI and VIP. In total, included studies randomised 7994 patients. Most studies used CCMD to verify the diagnosis. 15 studies used Western classifications only. All studies but one used the HAMD to assess depressive symptoms, and the majority of studies used the TESS to assess side-effects.

A total of 19 trials (20 comparisons) compared two SSRIs (fluoxetine, citalopram, escitalopram, fluvoxamine, paroxetine, or sertraline). 19 trials compared SSRI with TCA, eleven trials with TCM (9 trials with herbals, 1 trial with electrical acupuncture and 1 trial with manual acupuncture), five trials with a tetracyclic antidepressant (teCA), six with SNRI, four with norepinephrine reuptake inhibitor (NARI), four with norepinephrine and dopamine reuptake inhibitor (NDRI), and two with serotonin antagonist and reuptake inhibitor (SARI). Three placebo-controlled studies were retrieved. The median number of patients per study was 53 (range: 20–480).

#### Primary outcomes

**Figure 2:**
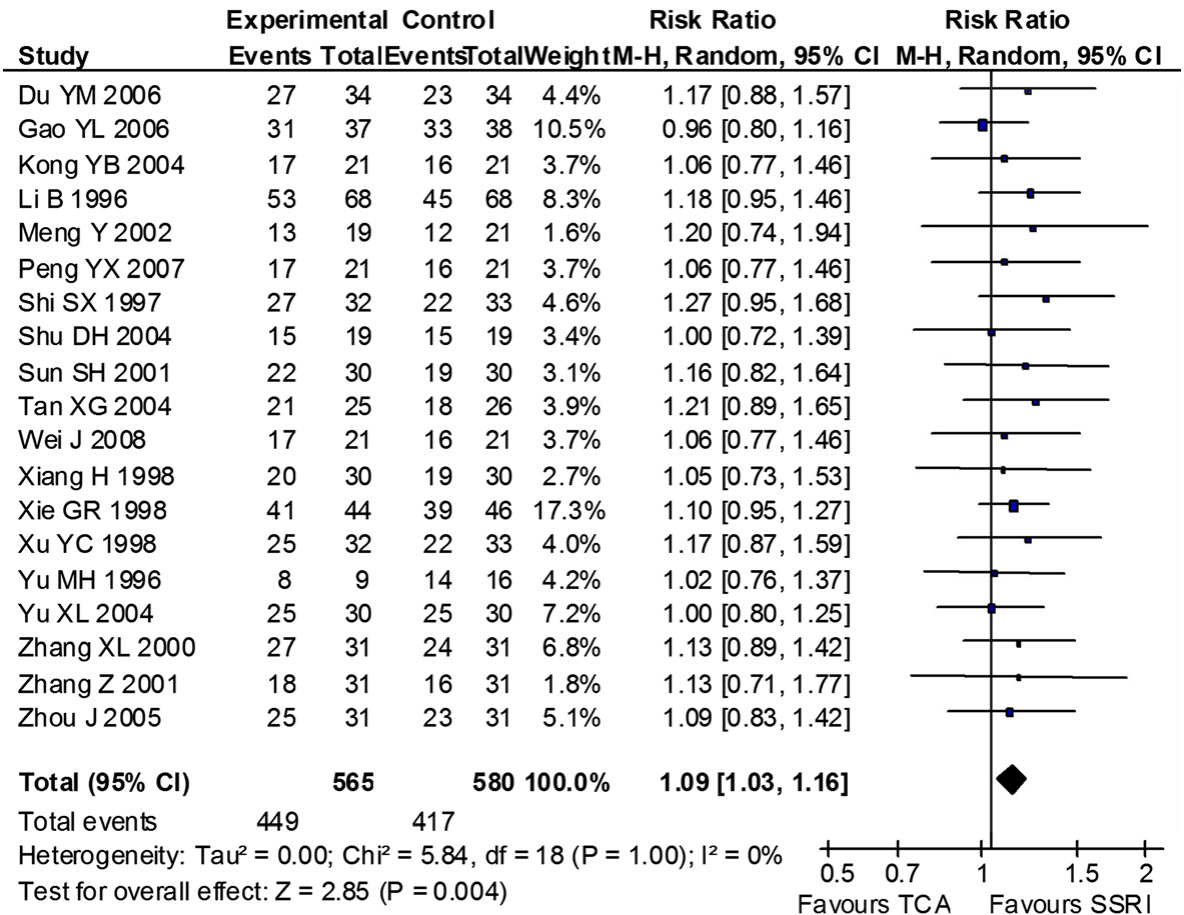
**Forest plot of comparison response rates, outcome SSRIs vs TCAs.** SSRI: Selective serotonin reuptake inhibitor. TCA: tricyclic antidepressant. M-H: Mantel-Haenszel. CI: confidence interval.

In terms of efficacy, SSRIs were statistically significant superior to TCA (response rate: MH RR 1.09, 95% CI 1.03 to 1.16; remission rate: MH RR 1.25, 95% CI 1.12 to 1.40, see Figure 2). No significant differences were observed regarding dropout rates due to side effects (Overview of results with no significant differences see Table 3). There was no evidence of heterogeneity (I^2^ = 0%), indicating that the effect sizes from the individual trials could be combined. Fluoxetine was statistically significant superior to placebo (response rate: MH RR 1.93 95% CI 1.48 to 2.52), although only one study was included for this comparison. All other comparisons showed no significant difference in the primary outcomes.

**Table 3:** **Overview of results with no significant differences**

**Remission**	**MH risk ratio (95%** **CI)**	**No. of comparisons**	**No. of subjects**
Fluoxetine vs. any other SSRIs	0.98 (0.83-1.17)	6	576
Citalopram vs. any other SSRIs	0.92 (0.80-1.05)	8	753
Sertraline vs. any other SSRIs	0.93 (0.71-1.24)	1	34
SSRI vs. TCM	0.98 (0.80-1.20)	4	995
SSRI vs. teCA	1.00 (0.78-1.27)	5	426
SSRI vs. NARI	1.03 (0.80-1.32)	3	363
SSRI vs. NDRI	1.39 (0.79-2.47)	2	140
SSRI vs. SARI	1.06 (0.80-1.39)	1	240
SSRI vs. SNRI	0.96 (0.85-1.08)	4	370
Geriatric SSRI vs. TCA	1.25 (0.89-1.75)	4	154
Geriatric SSRI vs. teCA	1.16 (0.87-1.55)	3	214
Non-geriatric SSRI vs. teCA	0.73 (0.47-1.11)	2	212
**Response**	**MH risk ratio (95**% **CI)**	**No. of comparisons**	**No. of subjects**
Fluoxetine vs. any other SSRIs	0.97 (0.89-1.06)	7	648
Citalopram vs. any other SSRIs	1.02 (0.95-1.10)	8	803
Sertraline vs. any other SSRIs	1.06 (0.82-1.37)	1	92
SSRI vs. TCM	1.04 (0.98-1.11)	7	2008
SSRI vs. teCA	1.02 (0.85-1.24)	5	426
SSRI vs. NARI	1.07 (0.92-1.23)	2	300
SSRI vs. NDRI	1.03 (0.89-1.18)	3	309
SSRI vs. SARI	1.03 (0.90-1.19)	1	240
SSRI vs. SNRI	0.96 (0.90-1.03)	5	614
Geriatric SSRI vs. TCA	1.09 (0.92-1.29)	4	154
Geriatric SSRI vs. teCA	1.07 (0.90-1.28)	3	214
Non-geriatric SSRI vs. teCA	0.90 (0.49-1.65)	2	212
**Dropout rates due to side effects**	**MH risk ratio (95**% **CI)**	**No. of comparisons**	**No. of subjects**
Fluoxetine vs. any other SSRIs	0.88 (0.27-2.79)	1	240
Citalopram vs. any other SSRIs	1.70 (0.65-4.45)	4	600
SSRI vs. TCA	0.51 (0.25-1.05)	9	590
SSRI vs. TCM	0.66 (0.17-2.52)	3	326
SSRI vs. teCA	0.60 (0.28-1.30)	2	194
SSRI vs. NARI	0.25 (0.03-2.20)	1	240
SSRI vs. Placebo	3.00 (0.13-71.00)	1	64
SSRI vs. SNRI	1.58 (0.21-12.20)	2	82
Geriatric SSRI vs. TCA	0.29 (0.05-1.79)	2	100
Non-geriatric SSRI vs. TCA	0.57 (0.26-1.23)	7	490
Geriatric SSRI vs. teCA	0.50 (0.05-5.23)	1	62
Non-geriatric SSRI vs. teCA	0.62 (0.27-1.39)	1	132

MH: Mantel-Haenszel.

CI: confidence interval.

SSRI: Selective serotonin reuptake inhibitor.

TCA: tricyclic antidepressant.

teCA: Tetracyclic antidepressant.

TCM: Traditional Chinese Medicine.

NARI: Norepinephrine Reuptake Inhibitor.

NDRI: Norepinephrine and Dopamine Reuptake Inhibitor.

SARI: Serotonin Antagonist and Reuptake Inhibitor.

SNRI: Selective Noradrenaline Reuptake Inhibitor.

#### Secondary outcomes

On the basis of overall dropout rates and total TESS scores significant differences favoring SSRIs over TCAs were observed. There were no significant differences between SSRIs and other classes of antidepressants on any outcome. A significant difference favoring citalopram over sertraline was observed (Hedges’g =0.40, 95% CI 0.03 to 0.78) based on mean total HAMD scores at endpoint. There was no evidence of heterogeneity, indicating that the effect sizes from individual trials could be combined.

#### Sensitivity analyses

Three pairs of publications [18] and [19], [20] and [21], [22] and [23] with striking similarities in both text and figures were excluded in sensitivity analyses, however, exclusion did not substantially affect the main findings.

#### Subgroup analysis

No significant differences were found in the four studies including elderly patients only (see Table 3).

#### Publication bias

Funnel plots for the analysis of primary efficacy and tolerability outcomes in SSRIs versus TCAs as well as the Begg adjusted rank correlation test and Egger regression approach did not reveal a significant publication bias.

#### Risk of bias

**Figure 3:**
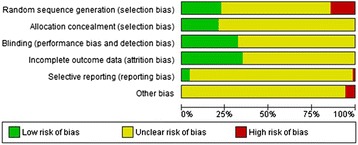
**Ratings of Risk of bias items presented as percentages across all included studies.**

The results for risk of bias in all studies included (and measured using the Cochrane risk of bias tool) are presented in Figure 3. The individual ratings for each study are presented in Additional file 1: Appendix 4. In general, reporting of articles published in Chinese journals was incomplete and inaccurate. In 16 (23%) studies adequate methods of sequence generation were described. On average 42% of 37 CONSORT checklist items (range: 16-81%) were reported. All authors failed to report trial registration. Informed consent of study participants was reported in 28 (39%) studies. 12 studies discussed results considering trial limitations or addressing sources of potential bias, and nine studies reported sources of funding. Abstract quality was also moderate at best. Both the terms “randomised” and “double-blind” were written only in titles and/or abstracts of 43 studies. Seven studies had no abstract, and 27 studies had no English abstract. 16 (22.5%) studies reported to use Intention-To-Treat-Analysis (ITT) analysis. 32 Studies (45.1%) failed to report dropout rates, whereas 15 studies reported dropout rates of less than 5%. Three trials, all published in English language journals, reported sample size calculation. Of the eleven studies comparing an antidepressant to TCM, seven reported to have applied a double-blind, double-dummy design. The risk of bias of these eleven studies was comparable to the risk of bias of the other studies.

**Figure 4:**
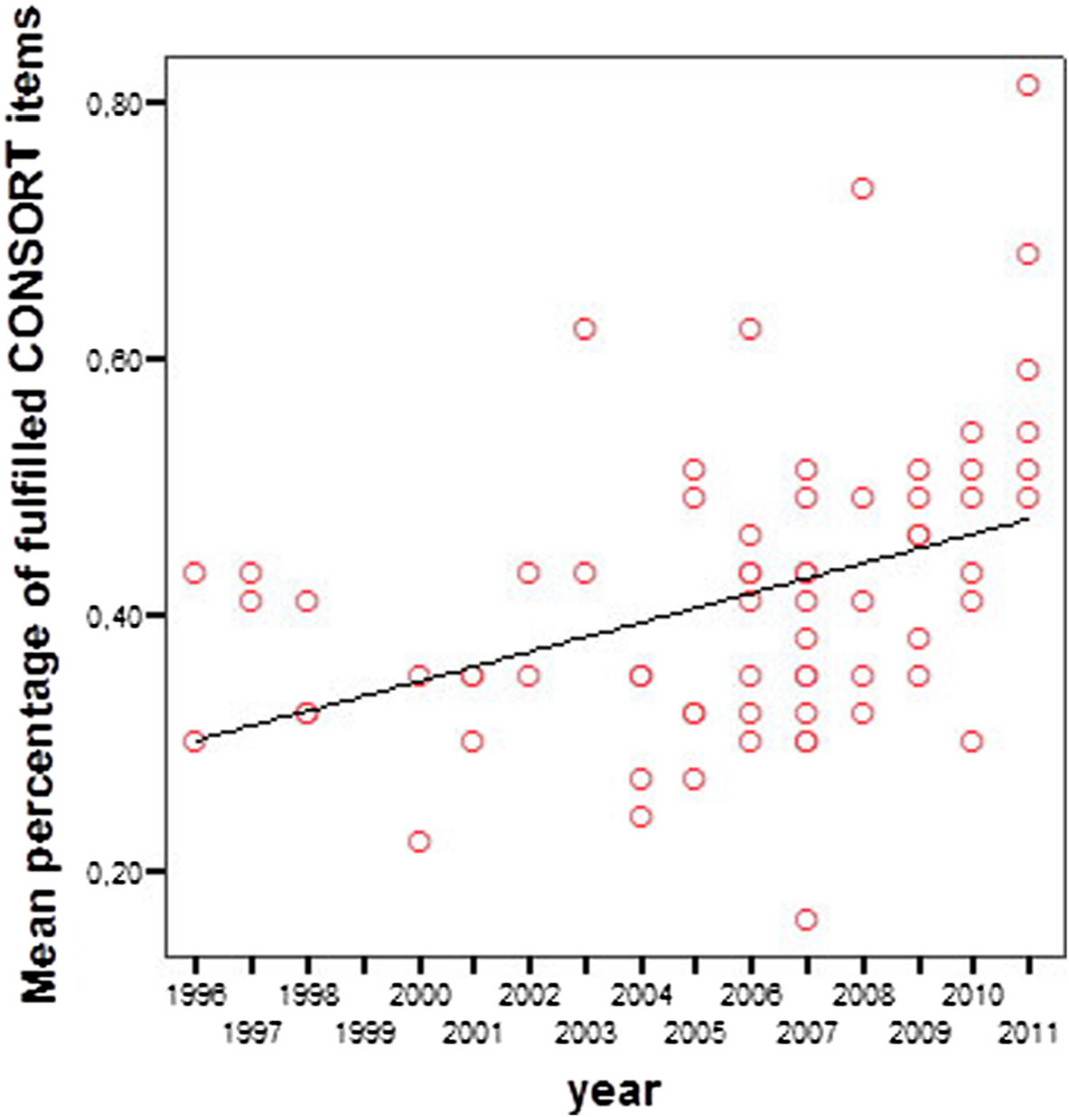
**Development of reporting quality using mean percentage of fulfilled CONSORT items.** CONSORT: Consolidated Standards of Reporting Trial.

Although conducted in different regions, three pairs of publications [18] and [19], [20] and [21], [22] and [23] showed striking similarities in both text and figures. In addition, another study [24] was found to have similarities with an excluded study [25]. Change over time of the reporting quality is presented in Figure 4, showing significant improvement in reporting quality as measured by the fulfilment of consort items (b = 0.397, p = 0.001).

## Discussion

### Summary of findings

Findings analyzed in this systematic review and meta-analysis provided evidence that SSRIs, in terms of response and remission, are slightly more effective than TCAs, which is inconsistent with most Western meta-analyses which have indicated comparable efficacy between SSRIs and TCAs [26–29], or showed that TCAs were more effective than SSRIs [30,31]. No significant differences were observed with respect to dropout rates due to side effects. This is not in line with evidence from Western trials suggesting superiority of SSRI over TCA in terms of tolerability and dropout rates [26–28]. TCA was associated with a higher prevalence of adverse effects in Drowsiness, Anxiety, Dry mouth etc. No significant differences were observed on any outcome between SSRIs and other antidepressant interventions such as SNRI, teCA, NARI, NDRI, and SARI (see Table 3). Like previous meta-analysis [32], the efficacy and safety of acupuncture therapy and herbs were comparable to SSRI. Different adverse effects were experienced by Chinese patients treated with acupuncture (needling pain, transient dizziness, and nausea) and with SSRI (headache, insomnia, and tiredness). There were no significant differences in effectiveness between different SSRIs, which confirmed the findings of Gartlehner et al. [33].

The findings confirmed deficiencies in the reporting quality of Chinese clinical trials as identified in other reviews [7–10]. Most studies were not large enough to provide accurate, generalizable results. The pairs of studies with striking similarities identified suggest instances of deficient practice of the peer review process in some Chinese scientific journals. However, in terms of the mean percentage of reported CONSORT items, a steady improvement of reporting quality was found in recent years.

### Limitations

Study limitations require consideration: First, the conclusions that can be drawn from this review are limited by the risk of bias and incomplete reporting of trials. We could not further determine associations of treatment effects with potential biases derived from methodological flaws. Due to the low reporting quality of abstracts, reading full text articles was the only way to identify potentially relevant studies. Second, only published and short-term studies were included. Although there were no restrictions on duration, we were not able to identify a long term study. Third, except for studies with a TCA as the comparator, the number of studies included for each comparison was small. Therefore, the lack of significant differences could be due to a lack of power.

## Conclusions

The present study systematically assessed the quality of Chinese double-blind RCTs in the treatment of depression, and it determined the efficacy and tolerability of SSRIs. In spite of the limitations mentioned, the results of this review shed light on the quality of Chinese medical articles and lead to several important recommendations for future research: First, CONSORT guidelines should be used by editors and researchers in China. Second, further effort is warranted to utilize the research resource in China by noticing the problem of the very low overlap of the Western and Chinese databases. Finally, given the widespread use of SSRIs and other antidepressants in China, this review shows a surprising lack of high quality evidence for the efficacy of these drugs in Chinese patients. The findings of a significant advantage of SSRI over TCA in terms of response rate and remission rate should be replicated by large high-quality Chinese studies. In Chinese populations the efficacy and safety of acupuncture therapy and herbs as monotherapy were comparable to SSRI, the first line treatment option of depressive illness. Therefore, the clinically differences in adverse reaction profiles may be the most important factor in clinical choice.

## Additional file

Additional file 1: Appendix 1. References to studies included. **Appendix 2**: References to studies excluded. **Appendix 3**: Characteristics of included studies. **Appendix 4**: Risk of bias of included studies.
