# Supplementary material for: A systematic review of Chinese randomized clinical trials of SSRI treatment of depression
Source: BMC Psychiatry. 2014 Aug 27;14:245. doi: 10.1186/s12888-014-0245-4 (PMC4149207; doi:10.1186/s12888-014-0245-4)
Supplement: Additional file 1: Appendix 1. — References to studies included. Appendix 2: References to studies excluded. Appendix 3: Characteristics of included studies. Appendix 4: Risk of bias of included studies. [file 12888_2014_245_MOESM1_ESM.docx]

Appendix 1: References to studies included

[1] Cai JY: **A comparative study of reboxetine and citalopram in the treatment of Depression.** *Journal of Psychiatry* 2007, **20**: 159-166.

[2] Cao HJ, An CF, Song XQ, Li Y, Li DZ: **A comparative study of bupropion in the treatment of Depression [Chinese]**. *Chin J Misdiagn* 2008, **8**: 3569-3570.

[3] Chang SH, Lin JZ, Chao GY: **A double blind comparative study of sertraline and maprotiline in the treatment of gerontism depression [Chinese*].*** *J Clin Psychol Med* 2006, **16**: 242.

[4] Chen EM: **Venlafaxine Retarder and Paroxitine for Refractory Depression: A Controlled Study.** *China Modern Doctor* 2010, **48**: 30-31.

[5] Chen LQ, Shi ZB, Li MJ, Sun H, Li XL, Dai EL, Yang XC, Li H, Peng XM, Zhang Y: **"An Shen Er Hao" treatment depression of clinical study.** *Journal of Chinese Modern Traditional Chinese Medicine* 2005, **1**: 101-105.

[6] Chen YH: **Comparative study of Escitalopram and Mianserin in the Treatment of Elderly Depression.** *Harbin medical journal* 2010, **30**: 30-33.

[7] Du B, Zhang HY, Huang SZ, Xie SP, Chen YG, Xu XF, Li HC, Zhang JP: **Efficacy and safety of Anjiaxin capsules in treatment of mild or moderate depression.** *Chinese Journal of New Drugs* 2007, **16**: 719-723.

[8] Du XS, Lu JK: **Double blind study of citalopram and sertraline in treatment of first-episode aged depression.** *J Clin Psychiatry* 2009, **19**: 196-197.

[9] Du XS, Wu XM, Zhang FM: **Double blind comparative study of citalopram and fluoxetine in treatment of depression.** *J Clin Psychol Med* 2007, **17**: 407-408.

[10] Du YM, Wu RQ, Wang JX: **A comparative study of fluoxetine and amitriptyline in the treatment of depressive disorder [Chinese].** *Hebei Medical Journal* 2006, **28**: 507.

[11] Fan HT: **A study of fluvoxamine in the treatment of post-stroke depression [Chinese].** *Chin J Geriatr Heart Brain Vessel Dis* 2007, **9**: 607.

[12] Fang LQ, Yang ZC: **A comparative study of citalopram and fluoxetine in the treatment of post-stroke depression [Chinese].** *Journal of harbin medical university* 2007, **41**: 73-74.

[13] Gao YL, Li WB, Wang HL: **A comparative study of fluoxetine and amitriptyline in the treatment of post-stroke depression.** *Medicl Journal of Chinese People's Health* 2006, **18**: 161-164.

[14] Han GL, Du XB, Song ZQ, Liu GL, Liu LX, Jian YL, Er HH, Li PS, Xu CH, Wang CY, Zhao XL, Xu HN, Wang RY, Li J, Zhang HW, Ye XL: **A clinical study of fluoxetine combined with Hong Jing Tian in the treatment of gerontism depression plateau [Chinese].** *Chinese Journal of Gerontology* 2006, **8**: 1017-1019.

[15] Han ZL, Guan NH, Zhang JP, Wen SL, Tao J: **A comparative study of sertraline, fluoxetine and paroxetine in the treatment of depression [Chinese].** *Chin Hosp Pharm J* 2002, **22**: 293-295.

[16] Hong CJ, Hu WH, Chen CC, Hsiao CC, Tsai SJ, Ruwe FJL: **A Double-blind, Randomized, Group-Comparative Study of the Tolerability and Efficacy of 6 Weeks' Treatment with Mirtazapine or Fluoxetine in Depressed Chinese Patients.** *J Clin Psychiatry* 2003, **64**: 921-926.

[17] Hsu Ju-Wei, Su Tung-ping, Huang Chen-Ying, Chen Ying-Sheue, Chou Yuan-Hwa: **Faster Onset of Antidepressant Effects of Citalopram Compared With Sertraline in Drug-NaBve First-Episode Major Depressive Disorder in a Chinese Population.** **A 6-Week Double-Blind, Randomized Comparative Study.** *Journal of Clinical Psychopharmacology* 2011, **31**: 577-581.

[18] Hu MR, Li LH, Lu XZ, Xun GL, Chen JD: **Escitalopram vs citalopram for depression: a randomized, double-blind, double-dummy, multicenter, parallel controlled study.** *Central South Pharmacy* 2010, **8**: 67-69.

[19] Huang P, Li ZR, Wang KY, Cheng B: **A double blind comparative study of citalopram in the treatment of post-stroke depression and impact of nerve function rehabilitation [Chinese].** *Chin J Nerv Ment Dis* 2006, **32**: 466-467.

[20] Jiang T, Wang CY, Chen YG, Xu XF, Wang GH, Yang GF, Zhai QM, Weng YZ: **A randomized, double-blind, controlled, multicenter clinical trial of Nefazodone Hydrochloride Tablets in the treatment of depression [Chinese].** *The Journal of Practical Medicine* 2010, **26**: 2614-2617.

[21] Jiang XY, Ren K: **A comparative study of escitalopram and citalopram in the treatment of depression [Chinese].** *Journal of Qiqihar Medical College* 2009, **30**: 45-46.

[22] Kong Qingmei, Shu Liang, Zhang Hongyan, Jiao Fuying, Han Zucheng, Wang Jian, Du Bo, Shi Jianguo, Wang Xiaoping, Ai Changshan, Gao Chengge, Li Lingzhi, Huang Lan: **Efficacy and safety of Morinda officinalis oligose capsule in the treatment of depression.** *Chin J Clin Pharmacol* 2011, **27**: 170-173.

[23] Kong YB, Song YP: **A clinical observation of citalopram in the treatment of post-stroke depression [Chinese].** *J Clin Psychol Med* 2004, **14**: 366-367.

[24] Li B, Wang ZX, Yao FC, Huang MS, Ma C, Sun XL: **A double blind controlled trial comparing sertraline and amitriptyline in major depression.** *Journal of Clinical Psychological Medicine* 1996, **6**: 329-331.

[25] Li GJ, Li HF, Shen XL, Yin ML, Zhou ZQ. **Bupropion SR in treatment of major depression: a randomized, double blind, double dummy and controlled study.** *Shanghai Archives of psychiatry* 2005, **17**: 160-162.

[26] Li HF, Ma C, Chen YG, Fan JX, Lan CA, Cheng NN, Gu NF: **Clinical effect of reboxetine in the treatment of anxiety in depressive patients.** *Chinese Journal of Clinical Pharmacy* 2007, **15**: 339-342.

[27] Li HF, Xie SP, Li M, Shi JA, Shen XL, Fan JX, Gu NF: **Study of Bupropion Hydrochloride Tablet and Fluoxetine in Treatment of Depression Multicenter Clinical Trial.** *Journal of Shanghai Jiaotong University (Medical Science)* 2006, **26**: 377-380.

[28] Li Hongjie, Zhong Baoliang, Fan Yinping, Hu Hongtao: **Acupuncture for post-stroke depression: a randomized controlled trial.** *Chinese Acupuncture & Moxibustion* 2011, **31**: 3-6.

[29] Li J, Meng HQ, Liu F: **Comparison of Mirtazapine and Paroxetine in the Treatment of Patients with Refractory Depression.** *Chinese Mental Health Journal* 2007, **21**: 878-880.

[30] Li J, Shen WW, Liu Y, Xu L, Liu SM, Kuang WA: **A randomized double blind active controlled trial of efficacy and safety of escitalopram in the treatment of depression [Chinese].** *Chin J Evid-based Med* 2006, **6**: 552-556.

[31] Li LJ, Chao FY, Xiao H, Liu XL, Zhou YY, Xu YT: **A Clinical Study on A Randomized, Double-blind Controlled of ShuYu Capsule in Patients with Vascular Depression.** *Chinese Journal of Experimental Traditional Medical Formulae* 2010, **16**: 220-223.

[32] Li N, Ji WD, Zhang DH, Li Y: **Efficacy and safety of reboxetine versus fluoxetine for the elders with depression.** *Chinese Journal of New Drugs* 2006, **15**: 1682-1684.

[33] Li N, Xu Z, Zhao XR, Xu XF: **A randomized double blind controlled trial of duloxetine in the treatment of depression [Chinese].** *Medicine and Pharmacy of Yunnan* 2007, **28**: 345-347.

[34] Li XX, Tao F, Wang X, Li J: **Clinical effect of escitalopram in the treatment of depression [Chinese].** *Pharmaceutical and Clinical Research* 2010, **18**: 70-72.

[35] Liu Song-shan, Chen Wei-yin, Liu Fu-You, Xue Jie, Liu Yuan-xin, Zhao Yan-ling, Meng Cui-xia, Ji Hai-Wang: **A phase III clinical trial of Kexinshu in treatment of mild or moderate depression with syndrome of liver stagnation-spleen deficiency.** *Chin J New Drugs Clin Rem* 2011, **30**: 107-110.

[36] Lu XJ, Ma XJ: **170 cases of venlafaxine in the treatment of depression [Chinese].** *China Pharmaceuticals* 2008, **17**: 74.

[37] Luo HC, Halbriech U, Shen Y, Meng FQ, Zhao XY, Liang W, Tan CX: **Comparative study of electroacupuncture and fluoxetine for treatment of depression.** *Chin J Psychiatry* 2003, **36**: 215-219.

[38] Lv Zhen-Lei, Zhang Yun-biao, Qi Shu-guang: **A randomized double-blind study of escitalopram and citalopram in treating major depression.** *J Clin Psychiatry* 2013, **23**: 37-38.

[39] Ma X, Yang JJ, Ma YB: **Therapeutic effect of the treatment of traditional chinese medicines on 42 cases of post-stroke depression.** *Medicl Journal of Chinese People's Health* 2007, **19**: 358-374.

[40] Mao PX, Cai ZJ, Zhang HY, Li J, Xie SP, Xu XF, Xiong P, Zhou XT, Jiang SB, Shi XD: **A randomized, double-blind, parallel controlled, multicenter clinical trial of reboxetine in the treatment of depression.** *Chin J New Drugs Clin Rem* 2010, **29**: 490-494.

[41] Mao PX, Tang YL, Jiang F, Shu L, Gu XL, Li M, Qian MC, Ma C, Mitchell PB, Cai ZJ: **Escitalopram in major depressive disorder: a multicenter, randomized, double-blind, fixed-dose, parallel trial in a chinese population.** *Depression and anxiety* 2008, **25**: 46-54.

[42] Meng Y, Du J, Wang P: **Double-blind comparision of sertraline and amitriptyline in treatment for senile depressive disorder.** *Journal of Xinxiang Medical College* 2002, **19**: 181-183.

[43] Ou HX, Zhang XB, Qiao HF, Fu Q, Ji QM: **A comparative study of venlafaxine ER in the treatment of depression [Chinese].** *J Clin Psychol Med* 2001, **11**: 105-106.

[44] Ou Jian-jun, Xun Guang-Lei, Wu Ren-Rong, Li Le-Hua, Fang Mao-Sheng, Zhang Hong-Geng, Xie Shi-Ping, Shi Jian-Guo, Du Bo, Yuan Xue-Qin, Zhao Jing-Ping: **Efficacy and safety of escitalopram versus citalopram in major depressive disorder: a 6-week, multicenter, randomized, double-blind, flexible-dose study.** *Psychopharmacology* 2011, **213**: 639-646.

[45] Peng YX, Dai GH: **A clinical comparative study of fluoxetine and amitriptyline in the treatment of post-stroke depression [Chinese].** *Journal of Chinese Modern Medicine* 2007, **4**: 259-260.

[46] Qu M, Tang QS, Fei QH, Hou XJ: **Tonify Kidney and Disperse the Depressed Liver - Energy to Treat the Depression of Renal Deficiency and Liver Stagnation Syndrome: A Randomized Controlled Clinical Study.** *Chinese Archives of Traditional Chinese Medicine* 2007, **25**: 2343-2346.

[47] Shi SX, Gu NF, Yao FC, Chen QB, Huang JZ, Yang XM, Sheng YR, Yuan XC, Qian DS, Zhang YH, Xu YF: **A double blind randomised study comparing paroxetine and amitriptyline in major depression.** *Journal of Clinical Psychiatry* 1997, **7**: 70-73.

[48] Shu DH, Zhang K, He H, Han P: **Control study of paroxetine and amitriptyline in treatment of aged depression.** *Modern Medicine Health* 2004, **20**: 311.

[49] Sun SH, Shi Q, Zhang LY: **Efficacy and side effects of fluoxetine and doxepin in depressed patients.** *Shandong Arch Psychiatry* 2001, **14**: 229-230.

[50] Sun XL, Huang MS, Tang XD, Yang HY, Yan J: **A randomized double blind controlled study on the efficacy of paroxetine and sertraline in treatment of depression.** *Chinese Journal of New Drugs* 1997, **6**: 167-170.

[51] Tan XG, Li HX, Du ZG, Wang Q, Zhao YX, Feng XP: **A double blind study of citalopram and amitriptyline in the treatment of aged depression.** *Shandong Arch Psychiatry* 2004, **17**: 202-203.

[52] Wang XQ, Zhang HY, Shu L, Du B, Jiao FY, Han ZC, Gao CG, Ai CS, Li LZ, Huang L: **Efficacy and safety of morinda officinalis oligose capsule in the treatment of mild or moderate depression.** *Chinese Journal of New Drugs* 2009, **18**: 802-843.

[53] Wei J, An Z: **A clinical observation of citalopram in the treatment of Post-strokedepressive disorder [Chinese].** *Journal of Qiqihar Medical College* 2008, **29**: 2984.

[54] Wu Y, Shen YF, Li HF, Sun XL, Xu XF, Gao CG, Gu NF: **Bupropion SR in the treatment of depression comorbid with anxiety: a randomized, double-blind clinical trial.** *Shanghai Archives of psychiatry* 2009, **21**: 285-288.

[55] Xiang H, Li JX, Du HY, Zhou XD: **A clinical controlled study of paroxetine and amitriptyline in the treatment of depression.** *Sichuan Mental Health* 1998, **11**: 7-9.

[56] Xiao JS, Zhang JJ, Huang CY, Huang HJ: **The treamtment effect of betel nut for Post-strokedepression [Chinese].** *Journal of Mathmatical Medicine* 2005, **18**: 444-445.

[57] Xie GR, Huang MS, Xu MT, Fan CH: **A double blind comparative study of efficacy and side effects of paroxetine in the treatment of depression [Chinese].** *Chin J Clin Pharmacol* 1998, **15**: 18-21.

[58] Xie SY, Du XS: **A Double-Blind Study of Sertraline and Fluoxetine in Treatment of First-Episode Depression.** *Occup and Health* 2008, **24**: 2741-2742.

[59] Xu YC, Li YD, Li RY: **A comparative study of sertraline and amitriptyline in the treatment of depression [Chinese].** *Journal of Clinical Psychosomatic Diseases* 1998, **4**: 193-195.

[60] Xun GL, Li LH, Zhao JP, Fang MS, Zhang HG, Xie SP, Shi JG, Du B: **Escitalopram vs citalopram in treatment of depression: a randomized, double-blind, double-dummy, multicenter, parallel controlled study.** *Chin J New Drugs Clin Rem* 2009, **28**: 263-267.

[61] You NX, Wang XL: **A comparative observation of efficacy of citalopram and fluoxetine in the treatment of depression [Chinese].** *Acta Academiae Medicinae Suzhou* 2000, **20**: 859.

[62] Yu MH, Yuan Z: **A double blind comparative study of fluoxetine and amitriptyline in the treatment of gerontism depression [Chinese].** *Hainan Medical Journal* 1996, **3**: 184-185.

[63] Yu XL, Lv HC: **An observation of efficacy of citalopram in the treatment of Post-stroke depression and nerve function defect [Chinese].** *China science and technology information* 2004, **17**: 94-95.

[64] Yuan Yanbo, Huang Xuebing, Shu Liang, Li Huafang, Wang Chuanyue, Wu Wenyuan, Zhao Jingping, Li Ming, Xu Xiufeng, Zhang Hongyan: **Duloxetine versus paroxetine in the treatment of major depressive disorder: a randomized double-blind multicentre controlled study.** *Chinese Journal of New Drugs* 2011, **20**: 334-338.

[65] Zhang XL, Xu YC: **Control study of paroxetine and amitriptyline treating depression.** *Journal of heze medical college* 2000, **12**: 23-25.

[66] Zhang YL, Zhou BQ, Gao DW: **A comparative study of citalopram and fluoxetine in the treatment of depression [Chinese].** *Journal of Baotou Medical College* 2007, **23**: 615-616.

[67] Zhang Z, Ma HX, Xu HC: **A comparative study of sertraline in the treatment of Post-strokedepression [Chinese].** *Chinese Journal of Behavioral Medical Science* 2001, **10**: 347.

[68] Zheng Yuansong, Wu Xiqiang, Guo Yanyang: **Double-blind, placebo-controlled trial of Nefazodone hydrochloride tablets in treatment of depression at random.** *China Modern Doctor* 2012, **50**: 86-87.

[69] Zhou J, Xu XY, Liu YL: **A comparative study of paroxetine and imipramine in the treatment of depression.** *Medical Journal of Chinese People's Health* 2005, **17**: 731-732.

[70] Zhou Ping, Chen Yunchun, Wang Huaihai, Guo Li, Zhang Yahong, Bai Yuanhan, Wang Huaning, Tan Qingrong: **Randomized, double-blind controlled study of Duloxetine enteric tables in treatment of depression.** Journal of Psychiatry 2012, **25**: 161-163.

[71] Zhu GK, Li HL, Sun DZ: **A double-blind comparative study of cipramil and maprotiline in the treatment of senile depression.** *Modern Medicine Health* 2005, **21**: 761-763.

Appendix 2: References to studies excluded

**Exclusion Reason 1. not double-blind**

[1] An CG: **A comparative study of efficacy between fluoxetine and venlafaxine in the treatment of depressive patients [Chinese].** *China foreign medical treatment* 2008, **24**: 70.

[2] Ayiguli AS, Peng YH, Wen LB: **An analysis of 120 cases of Jiaweixiaoyaosan in the treatment of Post-stroke depression [Chinese].** *Chin J MAP* 2009, **26**: 1196-1197.

[3] Bai HP: **A comparative study of venlafaxine and fluoxetine in the treatment of depression [Chinese].** *Medical Journal of Chinese People's Health* 2006, **18**: 1032-1033.

[4] Bai LD, Tan MG: **Comparison between venlafaxine and fluoxetine in the treatment of major depression with anxiety [Chinese].** *Chin J New Drugs Clin Rem* 2001, **20**: 119-121.

[5] Bai ZQ: **A clinical comparative study of fluoxetine and amitriptyline in the treatment of depression [Chinese].** *Sichuan Medical Journal* 1999, **20**: 254-255.

[6] Chen HZ, Chen SH, Li XS, Lin JH: **A Controlled Study of the Efficacy of Venlafaxin Capsules and Paroxetine Tablets in the Treatment of Depression [Chinese].** *Herald of Medicine* 2001, **20**: 488-489.

[7] Chen M, Zhang CZ, Zhang Z: **Cost-effectiveness analysis in the drug therapy of depressive patients [Chinese].** *Journal of Jining Medical University* 2009, **32**: 256-258.

[8] Chen SM, Zhang XL, Lin AJ, Feng GZ: **Observation of efficacy of Wangyoufang in the treatment of depressive patients between 30-50 years old [Chinese].** *Journal of Liaoning University of TCM* 2009, **11**: 92-94.

[9] Chen SQ, Gao HY: **Clinical controlled observation on depression treated by venlafaxine-xr tablets [Chinese].** *J Clin Psychiatry* 2009, **19**: 112-113.

[10] Chen W, Wang GF, Chen XH, Sheng YL, Zhu H: **Effects of paroxetine on function recovery in patients with post-stroke depression [Chinese].** *Chinese Journal of Clinical Rehabilitation* 2002, **6:** 2014-2015.

[11] Chen WL, Wang QX, Cai CH, Li BH: **Fluoxetine in treatment of depression associated with hypertension [Chinese].** *Chinese Journal of New Drugs* 2003, **12**: 384-386.

[12] Chen XJ, Lin ZX, Li JL, Zou XB: **Observation of efficacy of antidepressants in the treatment of post-stroke depression and nerve function rehabilitation [Chinese].** *Chinese Journal of Clinical Rehabilitation* 2002, **6**: 1289.

[13] Chen XZ: **A clinical comparative study of Olanzapine combined with fluoxetine in the treatment of depression [Chinese].** *Chin J Clin Healthc* 2009, **12**: 620-622.

[14] Chen Y, He WY: **76 cases of fluoxetine in the treatment of post-stroke depression [Chinese].** *Chinese Journal of Clinical Rehabilitation* 2004, **8**: 666.

[15] Chen YD, Zhou TX, Zhang SP: **A comparative study of prozac and amitriptyline in the treatment of depression [Chinese].** *Sichuan Mental Health* 1997, **10**: 98-99.

[16] Chen ZM, Zhang JH, Li ZW, Zhang HM: **Comparison of efficacy of venlafaxine and imipramine and sertraline in the treatment of depression [Chinese].** *Chin J New Drugs Clin Rem* 2001, **20**: 109-111.

[17] Chou KL, Lee PWH, Yu ECS, Macfarlane D, Cheng YH, Chan SSH, Chi I: **Effect of Tai Chi on depressive symptoms amongst Chinese older patients with depressive disorders: a randomized clinical trial.** *Int J Geriatr Psychiatry* 2004, **19**: 1105-1107.

[18] Deng BY, Ren MZ, Zhang LJ: **A comparative study of venlafaxine and paroxetine in the treatment of depression [Chinese].** *J Clin Psychol Med* 2007, **17**:120.

[19] Ding WX: **A clinical comparative study of venlafaxine and paroxetine in the treatment of depression [Chinese].** *Journal of Clinical Psychiatry* 2006, **16**: 359.

[20] Ding WX: **Comparison of efficacy of venlafaxine and fluoxetine in the treatment of depression [Chinese].** *Sichuan Mental Health* 2007, **20**: 46.

[21] Dong JG: **Observation of efficacy of 30 cases of low-dose olanzapine combined with fluoxetine in the treatment of depression [Chinese].** *Shandong Medical Journal* 2009, **49**: 99-100.

[22] Fan ZJ: **A control study of venlafaxine and citalopram in treatment of depression [Chinese].** *China Medical Herald* 2008, **7**: 69-70.

[23] Fang NJ: **Clinical observation of fluoxetine in the treatment of post-stroke depression [Chinese].** *Strait Pharmaceutical Journal* 2009, **21**: 139-140.

[24] Fu H, Lin CH, Lin HJ: **A comparative study of venlafaxine ER and paroxetine in the treatment of depression [Chinese].** *The Journal of Practical Medicine* 2009, **25**: 2068-2069.

[25] Fu SX, Wu SC, Xu L, Cheng P, Wang T, Guo WY: **Comparative Study between Duloxetine and Fluoxetine in the Treatment of First Episode Depression [Chinese].** *China Modern Doctor* 2009, **7**: 85-86.

[26] Gao JF, Tao M, Zhang P: **Clinical study of the compliance with antidepressants [Chinese].** *Shanghai Archives of Psychiatry* 2003, **15**: 143-145.

[27] Gao MX, Zhu HR, Qi G, Zhou XD: **Clinical observation of venlafaxine and paroxetine in the treatment of depression [Chinese].** *People's Military Surgeon* 2008, **51**: 97-98.

[28] Gao XJ: **Effects of fluoxetine on the depressive symptoms, self-care ability of daily life and neurological function in stroke patients [Chinese].** *Chinese Journal of Clinical Rehabilitation* 2005, **9**: 12-13.

[29] Gao XL: **A clinical comparative study of venlafaxine ER in the treatment of depression without response of SSRIs [Chinese].** *Medical Journal of Chinese People's Health* 2006, **18**: 437-438.

[30] Guo QY, Feng EY, Ren HQ: **A clinical control study of venlafaxine and paroxetine in the treatment of depression [Chinese].** *Medical Journal of Chinese Civil Administration* 2002, **14**: 354-355.

[31] Han Y, Wan YX, Ma ZW: **The curative effects of venlafaxine and fluoxetine in patients with senile depression [Chinese].** *J Clin Psychosom Dis* 2004, **10**: 9-10.

[32] Han YQ, Mi JL, Ji SM: **A comparative study of mirtazapine and fluoxetine in the treatment of post-stroke depression [Chinese].** *Herald of Medicine* 2009, **28**: 1285-1286.

[33] He WM: **Comparison of efficacy of duloxetine and fluoxetine in the treatment of depression combined with chronic pain [Chinese].** *Herald of Medicine* 2010, **29**: 730-732.

[34] He YQ, Ma SH: **Comparative study of Venlafaxine and Fluoxetine in the treatment of Depression [Chinese].** *Chinese Health Care* 2007, **18**: 1-2.

[35] Hu AQ: **The Study of Venlafaxine and Fluoxetine Treating senile patients with depressive disorder and without psychotic symptom [Chinese].** *Journal of Heze Medical Journal* 2003, **15**: 8-9.

[36] Hu GT, Tan XQ, Yang L, Wang TL: **A comparative study of venlafaxine and paroxetine in the treatment of depression [Chinese].** *Medical Journal of National Defending Forces in Southwest China* 2003, **13**: 383-384.

[37] Hu HX, Ji RG: **A comparative study of venlafaxine ER and paroxetine in the treatment of depression [Chinese].** *Xinjiang Medical Journal* 2008, **38**: 7-10.

[38] Huang HF, Chen YH, Zhang WW, Chen BR, Chen ZJ, Peng ZZ: **Clinical efficacy of mirtazapine and fluoxetine in the treatment of senile depression [Chinese].** *Asia-Pacific Traditional Medicine* 2009, **5**: 83-84.

[39] Huang HF, Chen YH, Zhang WW, Chen BR, Chen ZJ, Peng ZZ: **Clinical efficacy of mirtazapine and fluoxetine in the treatment of senile depression [Chinese].** *Asia-Pacific Traditional Medicine* 2009, **5**: 43-44.

[40] Huang JM, Niu QS: **Cost-effectiveness Analysis in Treatment of Depression with Three Drugs [Chinese].** *Herald of Medicine* 2005, **24**: 636-637.

[41] Huang JQ: **A comparative analysis of citalopram and fluoxetine in the treatment of senile depression [Chinese].** Chin J Misdiagn 2010, **10**: 2819-2820.

[42] Huang LL: **Comparison of therapeutic effects and side effects between fluoxetine hydrochloride and clomipramine in patients with vascular depression [Chinese].** *Chinese Journal of Clinical Rehabilitation* 2005, **9**: 226-228.

[43] Huang P, Li ZR: **A comparative study of venlafaxine in the treatment of post-stroke depression [Chinese].** *Nervous Diseases and Mental Hygiene* 2002, **2**: 162-163.

[44] Hwang JP, Yang CH, Tsai SJ: **Comparison study of venlafaxine and paroxetine for the treatment of depression in elderly Chinese inpatients [Chinese].** *Int J Geriatr Psychiatry* 2004, **19**: 189-190.

[45] Jia R, Chen XM, Zhi X, Gao FX: **A comparative study of prozac and amitriptyline in the treatment of depression [Chinese].** *Medical Journal of Chinese Civil Administration* 1998, **10**: 241.

[46] Jia W, Zhang XL, Zhang DB, Liu MY: **Effect of early intervention on recovery of motor function and recurrent stroke in patients with post-stroke depression [Chinese].** *Chinese Journal of Clinical Rehabilitation* 2005, **9**: 4-5.

[47] Jiang GQ: **A comparative study of paroxetine (made in china) and venlafaxine in the treatment of depression [Chinese].** *Chongqing Medical Journal* 2009, **38**: 780-781.

[48] Jiang JY, Chen BP, Li XF: **Clinical observation of efficacy of anti-anxiety of venlafaxine and Zoloft [Chinese].** *Journal of Qiqihar Medical College* 2003, **24**: 15.

[49] Jing YL, Wang XY, Sun Y, Sun P: **Comparative study of citalopram and venlafaxine in treatment of senile depression [Chinese].** *J Clin Psychol Med* 2007, **17**: 263-264.

[50] Kang R, Zhu S: **Control study of venlafaxine and SSRIs in the treatment of refractory depression [Chinese].** *J Clin Psychosom Dis* 2007, **13**: 135-137.

[51] Kong L, Yan H: **Clinical Observation on the Effect of Jieyu Mixture Combined with Fluoxetine for 50 Depression Patients [Chinese].** *Journal of Traditional Chinese Medicine* 2009, **50**: 699-701.

[52] Kou GM, Yu L, Lu YP: **Comparative study of venlafaxine and paroxetine in the treatment of depression [Chinese].** *China Prac Med* 2009, **4**: 46-47.

[53] Lei T, Xun ZY, Cao J: **Comparison of efficacy and safety between escitalopram and venlafaxin in the treatment of depression [Chinese].** *Journal of Psychiatry* 2008, **21**: 406-408.

[54] Li DS, Ju JX, Pang YD: **Comparative study of citalopram and venlafaxine in the treatment of depression [Chinese].** *J Clin Psychol Med* 2005, **15**: 158-159.

[55] Li L: **Clinical analysis of 30 cases of venlafaxine in the treatment of nonpsychotic depression [Chinese].** *Journal of Youjiang Medical College for Nationalities* 2007, **29**: 35-36.

[56] Li WQ: **Comparison of fluoxetine and amitriptyline in the treatment of post-stroke depression [Chinese].** *Chinese Journal of New Drugs and Clinical Remedies* 1998, **17**: 267-269.

[57] Li XF, Liu ZY, Zhang Y: **A comparative study of citalopram and fluoxetine in the treatment of depressive disorder [Chinese].** *J Clin Psychosom Dis* 2005, **11**: 352-353.

[58] Li XP, Ye H, Lu Q: **An economic analysis of antidepressants in the treatment of depression [Chinese].** *Strait Pharmaceutical Journal* 2008, **20**: 121-122.

[59] Li Y, Zhang XN, Wu ZM: **Comparative Study on the Effects of paroxetine and venlafaxine in treating depression [Chinese].** *Chinese Journal of Clinical Rehabilitation* 2004, **8**: 4174-4175.

[60] Li YM, An Y, Li SZ: **A comparative study of efficacy and the relationship with plasma concentration of venlafaxine in the treatment of depression [Chinese].** *Sichuan Mental Health* 2004, **17**: 134-136.

[61] Li ZH, Yuan YG: **A comparative study of venlafaxine and citalopram in the treatment of depression [Chinese].** *J Clin Psychol Med* 2007, **17**: 414.

[62] Liang SL, Cao SL, Xie WG: **A comparative study of fluvoxamine and fluoxetine in the treatment of senile depression [Chinese].** *The Journal of Practical Medicine* 2010, **26**: 1620-1622.

[63] Lin CH, Lin KS, Lin SY, Chen MC, Lane HY: **Time to Rehospitalisation in Patients With Major Depression Disorder Taking Venlafaxine or Fluoxetine [Chinese].** *J Clin Psychiatry* 2008, **69**: 54-59.

[64] Liu GJ, Wang LM, Pan SL, Zhang HX, Wang XG, Yu XP, Ren CX: **Clinical observation of efficacy of citalopram and fluoxetine in the treatment of depression [Chinese].** *Sichuan Mental Health* 2009, **22**: 36-37.

[65] Liu H, Liang TY: **A controlled Study in the Treatment of depression with venlafaxine and fluoxetine [Chinese].** *Shandong Arch Psychiatry* 2002, **15**: 81-83.

[66] Liu L, Li N: **Analysis of efficacy of bupropin in the treatment of senile depressive disorder [Chinese].** *Chin J Misdiagn* 2009, **19**: 1568-1569.

[67] Liu ML: **A comparative study of venlafaxine and fluoxetine in the treatment of depression [Chinese].** *Health Psychology Journal* 2000, **8**: 207-208.

[68] Liu W, Sun L: **Comparative studies on the efficacy of fluoxetine hydrochloride and clomipramine in the treatment of children's depression [Chinese].** *Tianjin Pharmacy* 2005, **2**: 25-26.

[69] Liu WT, Yang CG, Guo H: **A control study between venlafaxine und fluoxetine in the treatment of depression [Chinese].** *J Clin Psychosom Dis* 2004, **10**: 11-12.

[70] Liu XW, Zhang CL, Wu Y, Wu EN, Xu WL, Xu LF: **A comparative study of fluoxetine, venlafaxine and amitriptyline in the treatment of depression [Chinese].** *Nervous Diseases and Mental Hygiene* 2003, **3**: 52-53.

[71] Liu XW, Yang QP, Zhu PJ, Wang JL: **Study on the effects of duloxetine and fluoxetine on late-on set patients with depression [Chinese].** *Pract Geriatr* 2010, **24**: 162-164.

[72] Liu YH, Feng W, Xu MX: **A comparative study of vanlafaxine and fluoxetine in the treatment of post-schizophrenic depression [Chinese].** *Medical Journal of Chinese People Health* 2003, **15**: 327-329.

[73] Liu YM, Yu B, Li YF, Wan H: **A comparative study of venlafaxine and citalopram in the treatment of senile depression [Chinese].** *Journal of Clinical Psychiatry* 2007, **17**: 69.

[74] Lv WJ, Zhang ZF: **A comparative study of venlafaxine ER and citalopram in the treatment of post-stroke depression [Chinese].** *Chin J Mod Drug Appl* 2008, **2**: 33-34.

[75] Ma ZW, Li MX, Meng QW: **Curative Effect Comparasion of Venlafaxine and Fluoxetine in Treating Depression [Chinese].** *Chinese General Practice* 2004, **7**: 1335-1336.

[76] Min QX, Yang SH, Zhang TX: **Comparative study of clomipramine and fluoxetine in treatment of child depression [Chinese].** *Journal of Pediatric Pharmacy* 2008, **14**: 42-43.

[77] Min ZX: **A correlational study of citalopram and paroxetine in the treatment of depression [Chinese].** *Chinese community doctors* 2007, **14**: 28.

[78] Na WQ: **Control Comparison of Citalopramand Amitriptyline in Treating Post-stroke Depression. Anonymous [Chinese].** *Journal of Qiqihar Medical College* 2011, **28**: 906-908.

[79] Pan KY, Liu XY, Yang JZ, Zhu L, Wang XM, Yang SM: **Cost-effectiveness analysis of depression with paroxetine, venlafaxine and citralopram [Chinese].** *Chinese Journal of Clinical Rehabilitation* 2005, **9**: 16-18.

[80] Peng JF, Feng Z, Wei ZG: **A study of early improvement under Venlafaxine predicting later efficacy in patients with depression [Chinese].** *Medical Journal of Chinese People Health* 2009, **21**: 1228-1230.

[81] Peng Y: **A control study of venlafaxine vs fluoxetine in the treatment of elderly depression [Chinese].** *J Clin Psychosom Dis* 2008, **14**: 320-322.

[82] Qian MC, Sun JS, Liu JB: **A clinical study of venlafaxine in the treatment of treatment resistant depression [Chinese].** *Chin J Nerv Ment Dis* 2002, **28**: 130-131.

[83] Qin SP: **The efficacy and tolerability of venlafaxine and fluoxetine in treatment of patients with first-episode depression [Chinese].** *Medicl Journal of Chinese People's Health* 2006, **18**: 519-521.

[84] Qiu TW, Wang XH, Li ZX: **A comparative analysis of venlafaxine and paroxetine in the treatment of depression [Chinese].** *Medical Journal of Chinese People's Health* 2005, **17**: 285-286.

[85] Qiu Z: **Clinical Analysis of 189 Cases of Fluoxetine in the Treatment of Post-stroke Depression [Chinese].** *China Journal of Health Psychology* 2009, **17**: 907-908.

[86] Qu F, Cai XF, Gu YG, Zhou J, Zhang RJ, Burrows E, Huang HF: **Chinese Medicinal Herbs in Relieving Perimenopausal Depression: A Randomized, Controlled Trial.** *The Journal of Alternative and Complementary Medicine* 2009, **15**: 93-100.

[87] Qu HJ, Sun MJ, Peng HY, Wang L: **An analysis of efficacy and drug economic of venlafaxine and fluoxetine in the treatment of depression [Chinese].** *Herald of Medicine* 2004, **23**: 725-726.

[88] Ren K, Sun HM, Xie LY: **A comparative study of venlafaxine ER and paroxetine in the treatment of depression [Chinese].** *Journal of Qiqihar Medical College* 2009, **30**: 1173-1174.

[89] Shan PY, Liu SP, Chi ZF: **Effect of Fluoxetine on Treatment of post-stroke Depression [Chinese].** *Acta Academiae Medicinae Shandong* 2001, **39**: 229-233.

[90] Shen WY: **A comparative study of venlafaxine and paroxetine in the treatment of depression [Chinese].** *Medical Journal of Chinese People's Health* 2008, **20**: 2850.

[91] Shi XG, Dong WK, Wang GY, Xu JT: **Clinical Research on Reboxetine for Senile Depressive Disorder [Chinese].** *Evaluation and analysis of drug-use in hospitals of China* 2008, **8**: 614-616.

[92] Song CH, Ding XY, Song LH: **Effectiveness of fluoxetine and venlafaxine on the treatment of post-stroke depression [Chinese].** *Beijing Medical Journal* 2008, **30**: 163-164.

[93] Song L, Yu X, Guo P: **Comparative Study of Mirtazapine and Paroxetine in the Treatment of Depression in Childhood [Chinese].** *Chinese Journal of Health Psychology* 2005, **13**: 347-348.

[94] Song ZW, Li Y, Cai LR: **Comparative study of venlafaxine and fluoxetine in the treatment of depression [Chinese].** *Medical Journal of Chinese People Health* 2006, **18**: 739-740.

[95] Sun CY, Wang GH, Zhou T, Tian YL: **A comparative clinical study for venlafaxine versus fluoxetine in the treantment of depression [Chinese].** *Chinese Journal of new Drugs* 2005, **14**: 617-619.

[96] Sun GB: **Comparison of efficacy** **and tolerability of escitalopram and venlafaxine ER in the treatment of depression [Chinese].** *China Pharmaceuticals* 2010, **19**: 56-57.

[97] Sun HQ, Sun BM: **A comparative study of venlafaxine and fluoxetine in the treatment of senile depression [Chinese].** *Journal of Community Medicine* 2007, **5**: 3-4.

[98] Sun JT: **A control study on fluoxetine in the treatment of acute post-stroke depression [Chinese].** *Anhui Medical Journal* 2009, **30**: 440-442.

[99] Sun XW, Shi HJ: **Comparative study of Fluoxetine and Amitriptyline in the treatment of depression [Chinese].** *China Medical Herald* 2010, **7**: 63-64.

[100] Tan EY: **A clinical analysis of fluoxetine combined with amitriptyline in the treatment of treatment resistant depression [Chinese].** *Medical Information* 2010, **5**: 1250-1251.

[101] Tan W, Gong FZ, Wang Y, Wei QP, Xu XY: **Comparison of efficacy and safety between citalopram and venlafaxine in the treatment of depression [Chinese].** *Hainan Medical Journal* 2008, **19**: 16-17.

[102] Tian AJ, Li ZL, Jin JH, Zhao SJ: **A comparative study of venlafaxine ER and citalopram in the treatment of depression [Chinese].** *China Modern Doctor* 2009, **47**: 90-111.

[103] Tsoi WF, Tan CT, Kok LP: **Fluoxetine in the Treatment of Depression in Asian (Chinese and Indian) Patinets in Singapore [Chinese].** *Singapore Med J* 1995, **36**: 397-399.

[104] Wang DG, Chen HZ, Yin G, Li XS: **A comparative study of venlafaxine and fluoxetine in the treatment of depressive patients [Chinese].** *J Clin Psychol Med* 2001, **11**: 107-108.

[105] Wang FQ, Dong XR, Pan YX, Liu M: **Effect of Yukangning in the Treatment of Post-stroke depression and nerve function recovery [Chinese].** *Chinese Journal of Clinical Rehabilitation* 2003, **12**: 1225.

[106] Wang GP, Jie R: **A comparative study of venlafaxine and fluoxetine in the treatment of depression [Chinese].** *J Clin Psychol Med* 2004, **14**: 354.

[107] Wang GY: **A clinical observation of efficacy of fluoxetine in the treatment of post-stroke depression [Chinese].** *Chinese Journal of Practical Nervous Diseases Apr* 2008, **11**: 104-105.

[108] Wang HJ, Wu ZH: **A comparative study of citalopram and fluoxetine in the treatment of senile depression [Chinese].** *Journal of Qiqihar Medical College* 2006, **27**: 910-911.

[109] Wang HY: **Comparison of efficacy and side effects of citalopram and fluoxetine in the treatment of depression [Chinese].** *Shaanxi Medical Journal* 2010, **39**: 487-488.

[110] Wang HZ: **Observation of efficacy of fluoxetine in the treatment of post-stroke depression [Chinese].** *Medical & Pharmaceutical World* 2009, **11**: 24.

[111] Wang JG, Hu YZ, Qi FS: **Comparison of event - related potential P300 in the geriatric depression treated with venlafaxine or fluoxetine [Chinese].** *Shanghai Archives of Psychiatry* 2006, **18**: 94-97.

[112] Wang L: **A comparative observation of efficacy of venlafaxine and paroxetine in the treatment of depression [Chinese].** *Chinese Journal of Clinical Rehabilitation* 2005, **9**: 190.

[113] Wang LG, Liu YM, Wang DM: **A comparative analysis of efficacy and side effects of venlafaxine in the treatment of senile depression [Chinese].** *Sichuan Mental Health* 2005, **18**: 33-34.

[114] Wang LG, Wang XY, Li YF, Bi HL, Wang XY: **A comparative study of paroxetine and venlafaxine in the treatment of senile depression [Chinese].** *Journal of Clinical Psychiatry* 2007, **17**: 351.

[115] Wang LL, Hu L: **Observation of efficacy of venlafaxine and fluoxetine in the treatment of major depression [Chinese].** *Health Psychology Journal* 2002, **10**: 49-50.

[116] Wang Q, Zhou ZB: **A Comparative Study of Fluoxetine, Paroxetine and Venlafaxine in the Treatment of Depression [Chinese].** *Health Psychology Journal* 2003, **1**: 255-257.

[117] Wang RC, Liu XY, Wu T: **A comparative analysis of venlafaxine and sertraline in the treatment of depression [Chinese].** *Med J West China* 2007, **19**: 318-319.

[118] Wang XL: **A comparative observation of duloxetine and fluoxetine in the treatment of senile depression [Chinese].** *Sichuan Mental Health* 2009, **22**: 109-110.

[119] Wang YW, Yang LQ, Sun J: **A controlled study in the treatment of depression with citralopram and fluoxetine [Chinese].** *J Clin Psychol Med* 2004, **14**: 225-226.

[120] Wei HR: **A comparative study of citalopram and venlafaxine in the treatment of senile depression [Chinese].** *Hainan Medical Journal* 2009, **20**: 54-55.

[121] Wei SZ: **The controlled study on venlafaxine and fluoxetine in depression [Chinese].** *Nervous Diseases and Mental Health* 2006, **6**: 352-353.

[122] Wu CH, Jiang L, Zhang GC: **A Controlled Research of Paroxetine and Fluoxetine in Treating Depression [Chinese].** *China Modern Doctor* 2009, **47**: 85-86.

[123] Wu YS, Chen YC, Lu RB: **Venlafaxine vs. paroxetine in the acute phase of treatment for major depressive disorder among Han Chinese population in Taiwan [Chinese].** *Journal of Clinical Pharmacy and Therapeutics* 2007, **32**: 353-363.

[124] Xia YC, Chen DY, Yu JH: **Controlled observation of depression with delusion treated by new antidepressants [Chinese].** *J Clin Psychol Med* 2006, **16**: 287-288.

[125] Xian H, Tang QS, Zhao J: **Treatment of depression of liver-qi stagnation and spleen-deficiency type with therapy of soothing liver and invigorating spleen [Chinese].** *Journal of Beijing University of Traditional Chinese Medicine* 2008, **31**: 856-859.

[126] Xiao B, Xie WJ, Shi ZY: **A clinical control study of venlafax ine and fluoxetine in the treatment of senile depression [Chinese].** *Chin J of Behavioral Med Sci* 2005, **14**: 703-704.

[127] Xiao B, Xie WJ, Qiu KF, Huang FM, Shi ZY, Zhang F, Wang, GQ: **A comparative observation of venlafaxine and fluoxetine in the treatment of depression [Chinese].** *J Clin Psychol Med* 2004, **14**: 161-162.

[128] Xiao YG: **Venlafaxine extended release and sertraline in the treatment of depression-control Study [Chinese].** *China Modern Doctor* 2007, **45**: 71-72.

[129] Xie KP, Han Y: **A Comparative Study of Citalopram and Venlafaxine in the Treatment of Out-patients with Depression [Chinese].** *China Journal of Health Psychology* 2007, **15**: 260-261.

[130] Xing SM, Run BC: **A comparative observation of efficacy of venlafaxine in the treatment of depression [Chinese].** *Sichuan Mental Health* 2006, **19**: 28-29.

[131] Xu FL, Xu LQ: **Escitalopram vs. Fluoxetine for Depression: A Control Study [Chinese].** *China Pharmacy* 2009, **20**: 1084-1086.

[132] Xu HC, Wang XH, Gao L: **Clinic contrast study of Venlafaxine slow release tablets for treatment of depression [Chinese].** *Medical Journal of Chinese People Health* 2010, **22**: 1217-1218.

[133] Xu K, Li Z: **A study of efficacy of mirtazapine and fluoxetine in the treatment of senile depression combined with anxiety syndrome [Chinese].** *Medical Journal of Chinese People's Health* 2010, **22**: 565-591.

[134] Xu SQ, Cao J, Huang WW: **Comparison of escitalopram with fluoxetine in old depressive patients [Chinese].** *Chinese Journal of New Drugs* 2010, **19**: 208-210.

[135] Xu YH, Gong YP: **A comparative study of venlafaxine and fluoxetine in the treatment of senile depressive disorder [Chinese].** *Chinese Journal of Current Practical Medicine* 2006, **5**: 72.

[136] Xu YM, Zhang LY: **A comparative study of venlafaxine and fluoxetine in the treatment of depression [Chinese].** *Journal of Clinical Psychiatry* 2007, **17**: 295.

[137] Xue WS: **A comparative study of venlafaxine and sertraline in the treatment of depression [Chinese].** *Science and technology information* 2008, **12**: 309-311.

[138] Yan WL: **Efficacies of Escitalopram and Fluoxetine on Treatment of Major Depression [Chinese].** China Pharmacist 2009, **12**: 628-629.

[139] Yang CJ, Lu XB, Yang DY, Tong XS, Huang X: **Comparison at the same time of efficacy of citalopram and amitriptyline in the treatment of depression [Chinese].** *Chinese Journal of Clinical Rehabilitation* 2005, **9**: 219.

[140] Yang JH, Shen XH, Li L: **A randomized controlled trail of venlafaxine and paroxetine in patients with depression [Chinese].** *Chin J New Drugs Clin Rem* 2007, **26**: 848-851.

[141] Yang L, Rong PH: **30 cases of Jiaweixiaoyaosan combined with fluoxetine in the treatment of stagnation of liver qi type of post-stroke depression [Chinese].** *Shaanxi Journal of Traditional Chinese Medicine* 2009, **30**: 150-151.

[142] Yang QS: **Recent Developments on the Treatment of Pigmentary Degeneration of Retina with Acupuncture [Chinese].** *Guiding Journal of TCM* 2007, **13**: 51-52.

[143] Yang Z, Song JB, Yin JB: **A control study on venlafaxine vs. citaloparm in the treatment of depression [Chinese].** *J Clin Psychosom Dis* 2007, **13**: 131-132.

[144] Yang Z, Tan WZ, Song JB: **Comparative study between venlafaxine and paroxetine in treatment of depression [Chinese].** *Medical Journal of Chinese People Health* 2007, **19**: 442-443.

[145] Yao CB: **A comparison of Jieyutong and Fluoxetine for treatment of depression [Chinese].** *Medical Journal of Chinese People Health* **2010**, 22: 519-521.

[146] Yao JP, Chen L, Cha CX, Aike BE, Du XQ: **A clinical observation of venlafaxine in the treatment of cardiovascular depression [Chinese].** *Journal of Bingtuan Medicine* 2006, **2**: 12-13.

[147] Yao W, Fei JY, Liu XH: **A comparative study of venlafaxine and fluoxetine in the treatment of depression [Chinese].** *Medical Journal of Chinese People's Health* 2008, **20**: 1147-1148.

[148] Ye Q, Gu XY, Liu JF: **A clinical control study of venlafaxine and paroxetine in the treatment of depression in outpatients [Chinese].** *J Clin Psychol Med* 2002, **12**: 84-85.

[149] Yu J: **Cognitive psychotherapy and fluoxetine in the treatment of post-schizophrenia depression [Chinese].** *Chinese community doctors* 2008, **1**: 33.

[150] Yu J, Wang PL, Zhang GC, Hao ZP: **Controlled study of citalopramand venlafaxine in treatment of patients with senile depression [Chinese].** *J Clin Psychol Med* 2006, **16**: 92-93.

[151] Yu JX, Li DH, Zhu CY: **A comparative study of venlafaxine amitriptyline and fluoxetine in the treatment of depression [Chinese].** *Shandong Arch Psychiatry* 2004, **17**: 84-86.

[152] Yu WZ, Yu FY, Fu ZG: **Comparative Research on the Efficacy and Drug Reaction of Aged Depression using Venlafaxine and FIuoxetine [Chinese].** *International Chinese Neuropsychiatry Medicine Journal* 2005, **6**: 54.

[153] Yue DH, Zeng BT, Liu LH: **A comparative study of sertraline and fluoxetine in the treatment of depression combined with physical syndrome [Chinese].** *Journal of Psychiatry* 2009, **22**: 297-298.

[154] Zhang DJ: **A controlled study of venlafaxine and fluoxetine for retarded depression in 32 cases [Chinese].** *Chongqing Medical Journal* 2008, **37**: 502-503.

[155] Zhang F, Mao QJ: **Control Study on Duloxetine and Fluoxetine in the Treatment of Depression [Chinese].** *China Journal of Health Psychology* 2009, **17**: 1298-1299.

[156] Zhang FX, Gao H: **A control study on Venlafaxine and Citalopram in the treatment of depression [Chinese].** *Medical Journal of Chinese People Health* 2009, **21**: 441-443.

[157] Zhang H, Chen J, Wang GH: **Comparison of citalopram and venlafaxine for depression disorder [Chinese].** *Chinese Journal of Clinical Rehabilitation* 2005, **9**: 4-6.

[158] Zhang HW, Wang CY, Xu HN, Zhao XL, Dai QX, Li J, Du, XB, Song, ZQ, Han GL, Liu, GL, Li PS, Lin HH: **Clinical study of effect of fluoxetine combined with Chinese medicine or tibetan drugs in treating senile depression in plateau district [Chinese].** *CJITWM* 2006, **26**: 202-204.

[159] Zhang JS, Liu TQ, Zhao JP, Hao W, Xie GR, Su LY: **Control study of mirtazapine and fluoxetine in therapy of depression [Chinese].** *Chinese Journal of Clinical Rehabilitation* 2003, 7: 4102-4104.

[160] Zhang L: **A comparative study of venlafaxine and paroxetine in the treatment of depression [Chinese].** *Journal of Chinese Modern Medicine* 2007, **4**: 544-545.

[161] Zhang L: **A comparative study of venlafaxine and paroxetine in the treatment of depression [Chinese].** *Medical Journal of Chinese People's Health* 2009, **21**: 1560.

[162] Zhang Q: **The Control Study between Venlafaxine and Amitriptyline, Fluoxetine in the Treatment of Depression [Chinese].** *China Modern Doctor* 2008, **46**: 31-33.

[163] Zhang SJ, Wang MJ, Hu J: **Comparative Study of Venlafaxine and Paroxetine in the Treatment of Depression [Chinese].** *China Journal of Health Psychology* 2006, **14**: 644-645.

[164] Zhang XD, Tang J: **A comparative study of citalopram (made in China) and fluoxetine in the treatment of depression [Chinese].** *J Clin Psychosom Dis* 2007, 13: 259-260.

[165] Zhang YL, Zhang JH, Liang W: **A comparative study of venlafaxine extended release vs. paroxetine in treatment of depression [Chinese].** *Shanghai Archives of Psychiatry* 2003, **15**: 341-344.

[166] Zhang Z: **Comparison of therapeutic effects of venlafaxine and fluoxetine in the treatment of senile depression [Chinese].** *Shandong Arch Psychiatry* 2001, **14**: 97-98.

[167] Zhang ZQ, Yang XR, Wang XD: **A clinical control study of venlafaxine and fluoxetine in the treatment of depression patients [Chinese].** *Chinese Journal of Current Clinical Medicine* 2004, **2**: 695-696.

[168] Zhao FT, Xu SM, Zhang QH, Wang XL, Liu HH: **Citalopram versus venlafaxine for the improvement of post-stroke depression [Chinese].** *Chinese Journal of Clinical Rehabilitation* 2005, **9**: 12-13.

[169] Zhao HF, Zhu J, Ye YH: **Comparative study on venlafaxine extended release and citalopram in the treatment of depression [Chinese].** *Chinese Journal of Practical Nervous Diseases* 2008, **11**: 55-57.

[170] Zhao HH: **Comparative Trial of Venlafaxine versus Fluoxetine in the Treatment of Depression [Chinese].** *Practical Clinical Medicine* 2002, **3**: 56-57.

[171] Zhao HY, Zhao SH: **A control study of venlafaxine vs. fluoxetine in the treatment of post-stroke depression [Chinese].** *J Clin Psychosom Dis* 2008, **14**: 201-202.

[172] Zhao XM: **A clinical observation of 60 cases of fluoxetine in the treatment of post-stroke depression [Chinese].** *Journal of Qiqihar Medical College* 2009, **30**: 2154.

[173] Zheng L, Li WD: **Observation of efficacy of venlafaxine and paroxetine in the treatment of depression [Chinese].** *Chinese Journal of Clinical Rehabilitation* 2003, **7**: 3001.

[174] Zheng XB, Luo Z, Zhang XD, Yang LC, Li JL: **Efficacy of quetiapine combined with citalopram in the treatment of senile depression [Chinese].** *The Journal of Practical Medicine* 2009, **25**: 957-958.

[175] Zhou GP, Li D: **A comparative study of venlafaxine ER and fluoxetine in the treatment of depression [Chinese].** *China Modern Doctor* 2009, **47**: 68-89.

[176] Zhou GP: **A comparative study of venlafaxine ER and Fluoxetine in the treatment of depression [Chinese].** *China Hydropower Medicine* 2010, **10**: 9-11.

[177] Zhou MJ, Yao LQ: **The efficacy and tolerability of venlafaxine and fluoxetine in treatment of elderly patients with first-episode depression [Chinese].** *Chin J Psychiatry* 2005, **38**: 157-60.

[178] Zhou MY: **A comparative study of venlafaxine ER and fluoxetine in the treatment of depression [Chinese].** *J Chlin Psychol Med* 2006, **16**: 178.

[179] Zhou Q, Ye JH, Yan JZ, Yu XL: **A clinial observation of 41 cases of Jiaweibuyanghuanwu soup combined with fluoxetine in the treatment of post-stroke depression [Chinese].** *Fujian Journal of TCM* 2009, **40**: 13-14.

[180] Zhou ZH: **The curative effects of fluoxetine combined with psychotherapy in patients with post-stroke depression [Chinese].** *J Clin Psychosom Dis* 2003, **9**: 93-94.

[181] Zhu JD, Tan MF, Sun F: **Observation of efficacy of fluoxetine in the treatment of post-stroke depression [Chinese].** *Chinese Journal of Practical Nervous Diseases* 2010, **13**: 80-81.

[182] Zhu S, Zheng JZ: **A comparative study of fluoxetine and amitriptyline in the treatment of depression [Chinese].** *Henan Journal of Diagnosis and Therapy* 2000, **14**: 62-63.

[183] Zhu YP, Gu ZZ: **Comparison of antidepressive efficacy and side effects of mirtazapine, Chlorimipramine and fluoxetine [Chinese].** *The Journal of Practical Medicine* 2004, **20**: 318-319.

[184] Zhu YP, Zhou HJ: **Comparison of venlafaxine, Chlorimipramine and fluoxetine in the treatment of depression [Chinese].** *J Clin Psychol Med* 2002, **12**: 355-356.

**Exclusion Reason 2. Inclusion of bipolar patients**

[1] Bai HL, Zheng SJ, Xin LM, Chen YL, Ma J: **A controlled study on the treatment of depression with citalopram and imipramine [Chinese].** *Medical Journal of Chinese People's Health* 2010, **22**: 1385-1386.

[2] Chen H, Wang J, Han P, Su ZH: **A double-blind comparative study of fluoxtine (made in china) and amitriptyline in the treatment of depression [Chinese].** *Sichuan Mental Health* 1997, **10**: 165-166.

[3] Chen JD, Guo XF, Luo Q, Xun GL, Xue ZM, Li LH, Zhao, JP, Chen YG: **A randomized and double-blind clinical trial of reboxetine mesylate for treatment of depression [Chinese].** *Chinese Journal of New Drugs* 2006, **15**: 1679-1681.

[4] Chen JD, Guo XF, Li LH, Luo Q, Xue ZM, Zhao JP, Chen YG: **A randomized, double blind and positive control clinical trial of nefazodone for the treatment of depression [Chinese].** *Chinese Journal of New Drugs* 2006, **15**: 464-466.

[5] Cheng WH, Yan WW: **Clinical study of II phase of fluoxetine made in China [Chinese].** *Shanghai Archives of Psychiatry* 1995, **2**: 99-103.

[6] Cheng WH: **Clinical study of II phase of fluoxetine made in China [Chinese].** *Shanghai Archives of Psychiatry* 1995, **7**: 89-116.

[7] Ding BK, Qin XX, Li XB, Wang Y, Xue HY, Liu XL: **A double blind controlled study of depression treated with sertraline and fluoxetine [Chinese].** *Journal of Clinical Psychiatry* 1998, **8**: 72-73.

[8] Dong HX: **Supportive therapeutic function of quetiapine in the treatment of refractory depression [Chinese].** *Medical Journal of Chinese People's Health* 2010, **22**: 1.

[9] Du B, Gao CG, Wang G, Xie SP, Xu XF, Tan QR, Jia KQ: **Efficacy and safety of duloxetine with fluoxetine in the treatment of major depressive disorder [Chinese].** *Chin J Clin Pharmacol* 2009, **25**: 99-103.

[10] Du GP, Zhou GQ, Sun QY, Kou JH: **Comparison of mirtazapine and citalopram in female patients with climacteric depression [Chinese].** *China Journal of Health Psychology* 2010, **18**: 780-781.

[11] Duan YY, Yan K, Zhao HQ, Shi JA, Sun J: **Clinical study of II phase of fluoxetine made in China [Chinese].** *Shanghai Archives of Psychiatry* 1995, **2**: 1-3.

[12] Fan JX: **Randomized, multicenter, double blind comparative trial of reboxetine in the treatment of depression with or without anxiety [Chinese].** *Chin J Clin Pharmacol* 2008, **24**: 392-395.

[13] Fang YR, Wang ZC, Sheng JH, Xie B, Yuan ZM, Gao ZS, Shi QL, Bi YY: **A double-blind comparative test of fluoxetine and amitriptyline in the treatment of 105 cases patients with depressive disorders [Chinese].** *New Drugs and Clinical Remedies* 1997, **16**: 254-256.

[14] Gao CG, Wang G, Xu XF, Xie SP, Tan QR, Du B, Cheng NN, Wang W, Chen C, Fu W, Yang XB, Kang WH, Li Q, Ma XC: **Multi-center, randomized and double-blind controlled clinical trial of duloxetine enteric capsule in treatment of depression [Chinese].** *Chin J New Drugs Clin Rem* 2008, **7**: 481-485.

[15] Gao XH, Du CH, Wang LL: **A comparative study of paroxetine combined with olanzapine in the treatment of refractory depression [Chinese].** *Medical Journal of Chinese People's Health* 2009, **21**: 2966-2968.

[16] Gu NF, Li HF, Shu L, Zhang HY, Weng Z, Zhang XB, Ou HX, Zhou ZQ: **A multi-center, double-blind, randomized, parallel controlled clinical study of venlafaxine ER in the treatment of depression [Chinese].** *Chin J New Drugs Clin Rem* 2002, **21**: 66-71.

[17] Guo JX, Du WJ, Wang XL, Li T, Xu GY: **A control study of bupropion sustained-release in the treatment of depression [Chinese].** *J Clin Psychosom Dis* 2007, **13**: 323-324.

[18] Guo SW, Ou HX, Zhang XB, Sui MX, Yi TJ: **Comparative study on depression treated by tianeptine or fluoxetine [Chinese].** *Medical Journal of Chinese People's Health* 2007, **19**: 750-752.

[19] He TP, Liang JN: **A comparative analysis of paroxetine and amitriptyline in the the treatment of depression [Chinese].** *Anhui Medical and Pharmaceutical Journal* 1998, **2**: 17-18.

[20] He WZ: **A comparative study of sertraline and chlorimipramine in the treatment of depression [Chinese].** *Modern Journal of Integrated Traditional Chinese and Western Medicine* 2008, **17**: 2790-2791.

[21] Hong ZX: **A clinical observation of citalopram in the treatment of senile depression [Chinese].** *J Clin Psychiatry* 2009, **19**: 334.

[22] Hu YL: **Comparison study of citalopram and amitriptyline in the treatment of senile depression [Chinese].** *Journal Psychiatry* 2007, **20**: 39-40.

[23] Huang X, Xu MT, Lu Y, Huang XM: **A double blind comparative study of paroxetine in the treatment of depression [Chinese].** *Chinese Journal of Nervous and Mental Diseases* 1997, **23**: 93-97.

[24] Huang XJ, Gong ME, Tang ZY, Su C: **Comparison of mirtazapine and paroxetine in patients with first-episode climacteric depression [Chinese].** *Chinese Mental Health Journal* 2007, **121**: 428-430.

[25] Huo XP, Liu HY, Zhao SX, Li YH, Lv GR: **A double-blind comparative study of vitamin B12 in the accessory treatment of depression [Chinese].** *J Clin Psychol Med* 2007, **17**: 189-190.

[26] Jiang RH, Zhang HY, Shu L, Du B, Li HF, Ma C, Liu ZC: **A phase II randomized, double blind, multi-centers and parallel control clinical trial for bupropion SR in the treatment of depressive disorders [Chinese].** *Chinese Journal of New Drugs* 2006, **15**: 128-131.

[27] Jiang YH, Weng Z, Lv MS, Zhang SJ: **A controlled study in the treatment of depression with venlafaxine extended release and fluoxetine [Chinese].** *Shandong Arch Psychiatry* 2002, **15**: 199-200.

[28] Li GH, Yao HX, Yan SM: **Clinical study of II phase of fluoxetine made in China [Chinese].** *Shanghai Archives of Psychiatry* 1995, **7**: 112-114.

[29] Li HF, Zhao JP, Kuang WA, Yao PF, Chen JD, Sun XL, Gu NF: **A multi-center, double-blind, double-dummy, randomized, clinical study of Bupropion Hydrochloride Sustained- Release Tablets in the treatment of 72 cases with depression [Chinese].** *Chin J New Drugs Clin Rem* 2005, **24**: 614-618.

[30] Li HZ, Zhang YL, Zhang YL, Li MZ: **Double-blind study of fluoxetine augmented with olanzapine in the treatment of treatment-resistant depression [Chinese].** *Shandong Arch Psychiatry* 2006, **19**: 85-86.

[31] Li HZ, Zhang YL, Mu JL, Zhang YL: **Venlafaxine extended release and lithium salt for major depressive disorder in nonresponders to selective serotonine reuptake inhibitors [Chinese].** *Chinese Journal of Clinical Rehabilitation* 2006, **10**: 4-6.

[32] Li LH, Zhang HG, Chen JD, Zhao JP, Chen XG, Chen YG: **A control study on the curative effect and reliability of reboxetine with fluoxetine for treatment of depression [Chinese].** *Chin J of Behavioral Med Sci* 2006, **15**: 721-722.

[33] Li T, Wang Y: **A comparison analysis on the efficacy of fluvoxamine combined with ziprasidone in the treatment of refractory depression [Chinese].** *Tianjin Pharmacy* 2009, **21**: 30-32.

[34] Li T, Ma C: **A randomized and double-blind controlled clinical trial of reboxetine for treatment of depression [Chinese].** *International Medicine & Health Guidance News* 2004, **10**: 173-175.

[35] Li XC, Tang W: **Comparative observation on efficacy of suganjieyu capsules and citalopram hydrobromide tablets in treatment of mild and moderate-grade depression [Chinese].** *Practical preventive medicine* 2010, **17**: 328-330.

[36] Li YJ: **A clinical comparison study of venlafaxine ER and fluoxetine in the treatment of depression [Chinese].** *Chinese Journal of Coal Industry Medicine* 2008, **11**: 13-15.

[37] Liu DG, Li D: **Observation of 49 cases of effect of citalopram in the treatment of depressive disorder [Chinese].** *Journal of Community Medicine* 2007, **5**: 30-31.

[38] Liu P, Shu L, Lin K, Gu NF, Chen WL: **Comparative study of fluvoxamine and imipramine in the treatment of depression [Chinese].** *Chinese Journal of Nervous and Mental Diseases* 1998, **24 suppl**: 72-74.

[39] Liu Q, Zhang HY, Liu PL, Li ZJ, Xie SP, Gao CG, Xu XF, Du B, Tang MQ, Shen JQ, Li M, Zhang XB, Zhang Y, Shu L: **Randomized, multicenter, double blind comparative trial of Jin-yu-kang capsule in the treatment of mild and moderate depression [Chinese].** *Chin J Clin Pharmacol* 2007, **23**: 251-254.

[40] Liu S, Tian B: **A comparative study of escitalopram and venlafaxine in the treatment of Depression [Chinese].** *Journal of Psychiatry* 2008, **21**: 271-272.

[41] Liu YH, Xu MX: **A comparative study of paroxetine and amitriptyline in the treatment of senile melancholia [Chinese].** *Journal of Linyi Medical College* 2002, **24**: 321-323.

[42] Ma XC, Gao CG, Tan QR, Xu XF, Chu ZH, Zhang ML, Yu H: **Bupropion SR and fluoxetine in treatment of depression in multicenter clinical trial [Chinese].** *Journal of Xi'an Jiaotong University (Medical Sciences)* 2007, **28**: 533-537.

[43] Pan GY: **40 cases of venlafaxine in the treatment of depression [Chinese].** *China Pharmaceuticals* 2008, **17**: 63.

[44] Qin AL, Si GM, J M: **A clinical comparative study of mirtazapine and paroxetine in the treatment of depression [Chinese].** *Medical Journal of Chinese People's Health* 2008, **20**: 1561-1674.

[45] Sheng JH, Shen WL, Gao ZS, Yuan ZM, Wang ZC**: A double-blind controlled trial of 16 cases of fluoxetine and amitriptyline in the treatment of patients with depressive disorder [Chinese].** *New Drugs and Clinical Remedies* 1997, **16**: 21-22.

[46] Shi Y, Wang RJ: **A double-blind comparative study of prozac in the treatment of depression [Chinese].** *Shaanxi Medical Journal* 2002, **31**: 701-703.

[47] Song HB: **A clinical comparative study of citalopram and maprotiline in the treatment of depression [Chinese].** *Chinese Remedies & Clinics* 2008, **8**: 561-562.

[48] Sun H: **Analysis of efficacy of the traditional Chinese medicine and western medicine in the treatment of senile depression [Chinese].** *Journal of practical traditional Chinese medicine* 2010, **26**: 526-527.

[49] Sun ZZ, Li YF: **Comparative analysis of fluoxetine and imipramine [Chinese].** *Qingdao Medical Journal* 1997, **29**: 49.

[50] Tan MG, He Q, Gao CN, Ren YP, Zhang TL: **A double-blind comparative study of 18 cases of fluoxetine and amitriptyline in the treatment of patients with depressive disorder [Chinese].** *New Drugs and Clinical Remedies* 1997, **16**: 26-27.

[51] Wang JC, Xiong P, Xu XF, Ou YH: **Clinical observation of II phase of reboxetine in the treatment of depression [Chinese].** *Medicine and Pharmacy of Yunnan* 2007, **28**: 17-22.

[52] Wang JC, Xiong P, Ou YH, Li WY, Xu XF: **Clinical study of II phase of Bupropion Hydrochloride Sustain-release Tablet in the treatment of depression [Chinese].** *Medicine and Pharmacy of Yunnan* 2006, **27**: 638-542.

[53] Wang JC, Xiong P, Yang BC, Xu XF: **The third phase clinical trials of jinyukang for the treatment of mild to moderate depressive disorders [Chinese].** *Journal of Kunming Medical University* 2008, **1**: 110-115.

[54] Wang JX, Zhong XS: **A double-blind comparative study of fluoxetine and Imipramine in the treatment of depression [Chinese].** *Journal of Zhejiang Medical University* 1997, **26**: 135-137.

[55] Wang JX: **The synergetic effect of Sulpiride in the treatment of depression [Chinese].** *J Clin Psychiatry* 2010, **20**: 202-203.

[56] Wang KM, Bao LY: **A comparative study of fluoxetine and amitriptyline in the treatment of depression [Chinese].** *Shanghai Archives of Psychiatry* 1998, **10**: 171-172.

[57] Wang KY, Gu QC, Zhang XQ: **A double-blind comparative study of 16 cases of fluoxetine and amitriptyline in the treatment of patients with depressive disorder [Chinese].** *New Drugs and Clinical Remedies* 1997, **16**: 24-25.

[58] Wang SY: **Comparison of effect of venlafaxine and fluoxetine in the treatment of depressive episode [Chinese].** *Chinese Journal of Trauma and Disability Medicine* 2010, **18**: 80.

[59] Wang X, Zhang B, Li J, Sun XL: **A double-blind double dummy randomized controlled trial of bupropion hydrochloride sustain-release tablet in the treatment of depression [Chinese].** *Chin J Evid-based Med* 2007, **7**: 409-414.

[60] Wang XL, Li T, Ma C, Wen QQ: **A double-blind study of neurostan in the treatment of depression [Chinese].** *J Clin Psychol Med* 2003, **13**: 150-151.

[61] Wang XQ, Pei GX, Zhang YL, Zhou HX: C**omparative study between Magnesium valprote sustained release tablets with Sertraline in the treatment of refractory depression [Chinese].** *Medical Journal of Chinese People's Health* 2010, **22**: 2321-2324.

[62] Wang Y, Lei T, Zhang GS, Lv H: **A comparative study of citalopram and paroxetine in the treatment of senile depression [Chinese].** *Tianjin Med J* 2008, **36**: 895-896.

[63] Wang Y: **Adjuvant therapy of aripiprozole in the treatment of psychotic depression [Chinese].** *J Clin Psychiatry* 2009, **19**: 203-204.

[64] Wen YG, Chen WJ, Wang ZZ, Lai LQ: **A cost-efficacy analysis of three antidepressants in the treatment of depression [Chinese].** *China Pharmaceuticals* 2003, **12**: 68-69.

[65] Wu XQ, Yang LQ, Lu C, Xiong YY, Liu GX, Ma WT, Du HX: **Comparison paroxetine and doxepin in depressed patients with a double-blind randomized study patients [Chinese].** *Pharm J Clin Pla* 2000, **16**: 20-22.

[66] Xie GR, Su LY, Chen FH, Fan CH, Yu SY: **A double-blind comparative study of sertraline in the treatment of depression [Chinese].** *Chinese Journal of Nervous and Mental Diseases* 1998, **24 Suppl**: 80-82.

[67] Xie GR, Su LY, Wang CY, Yang ZW, Guo TS: **Clinical study of II phase of fluoxetine made in China [Chinese].** *Shanghai Archives of Psychiatry* 1995, **2**: 103-106.

[68] Xu ZP, Song ZW: **A comparative study of escitalopram and fluoxetine in the treatment of depression [Chinese].** *Jilin Medical Journal* 2009, **30**: 996-998.

[69] Yang XM, Deng HX: **A comparative study of domestic mirtazapine and paroxetine for depression in 25 cases [Chinese].** *China Pharmaceuticals* 2008, **17**: 53-54.

[70] Yao GZ, Liu P, Shu L, Xuan MZ, Wang ZH: **Citalopram in the treatment of depressive disorders--a multicenter open-label study [Chinese].** *Chinese Mental Health Journal* 1999, **13**: 162-164.

[71] Yue DH, Jiang ZL, Zhuang LM, Gao ZB, Liu LH: **A double-blind comparative study of 16 cases of fluoxetine and amitriptyline in the treatment of patients with depressive disorder [Chinese].** *New Drugs and Clinical Remedies* 1997, **16**: 27-28.

[72] Zhang HN, Hu SY, Li YH, Zhang CH: **Clinical observation of Baisong tablets for depression [Chinese].** *Journal of TCM Univ. of Hunan* 2008, **28**: 48-50.

[73] Zhang HY, Liu Q, Liu PL, Li ZJ, Xie SP, Gao CG, Xu XF, Zhang Y, Shu L: **Efficacy and safety of Jinyukang capsules in the treatment of mild and moderate depression [Chinese].** *Chinese Journal of New Drugs* 2006, **15**: 903-907.

[74] Zhang J, Xu SM, Zhao FT: **Efficacy of venlafaxine XR substituting selective serotonin reuptake inhibitors in treatment of major depression [Chinese].** *J Clin Psychol Med* 2007, **17**: 46-47.

[75] Zhang JH, Jiang XQ, Sun SH: **Observation of effect of fluoxetine and doxepin in the treatment of depression [Chinese].** *Chin J Pharmacoepidemiol* 2011, **12**: 119-121.

[76] Zhang JY, Wang JJ, Liu LF: **Comparative study of fluoxetine and amitriptyline in the treatment of senile depression [Chinese].** *Herald of Medicine* 1997, **16**: 205-206.

[77] Zhang YQ, Li GL, Lian EY, Ji YP: **A control study of sertraline and amitriptyline in treatment of aged depression [Chinese].** *J Clin Psychol Med* 2000, **10**: 212-213.

[78] Zhao BQ: **A comparative study of citalopram and venlafaxine in the treatment of depression [Chinese].** *Medical Journal of Chinese People's Health* 2008, **20**: 405-407.

[79] Zhao HY, Li XY, Sun ZK: **A comparative study of citalopram angument with olanzapine in the treatment of refractory depression [Chinese].** *Journal of Psychiatry* 2008, **21**: 370-372.

[80] Zhao HY: **Controlled study of escitalopram and venlafaxine in treatment of depression [Chinese].** *J Clin Psychiatry* 2008, **18**: 343-344.

[81] Zhao JG: **Effect of UBIO combined with fluoxetine in the treatment of senile depression [Chinese].** *Chinese Journal of Practical Nervous Diseases* 2008, **11**: 114.

[82] Zhao TH, Zhao H: **Comparison of effect of citalopram and fluoxetine in the treatment of depression [Chinese].** *Sichuan Mental Health* 2007, **20**: 53.

[83] Zhao ZL, Xian YP, Zhou HH: **A controlled trail of venlafaxine and paroxetine in treatment-refractory depression [Chinese].** *J Clin Psychol Med* 2003, **13**: 26-27.

[84] Zheng Y, Zhang LF: **Comparative Study of Citalopram and Venlafaxine in the Treatment of depression [Chinese].** *Medical Journal of Chinese People Health* 2008, **20**: 2762-2763.

[85] Zhong XS, Wang JX, Cheng RY, Li SL: **Clinical study of II phase of fluoxetine made in China [Chinese].** *Shanghai Archives of Psychiatry* 1995, **7**: 106-107.

[86] Zhou ZH, Yuan GZ, Zhu HM, Wang GQ: **Accessorial effects of quetiapine in the treatment of senile depression [Chinese].** *J Clin Psychiatry* 2008, **18**: 269-270.

**Exclusion Reason 3. Augmentation therapies**

[1] Duan WG, Zhu JS, Zeng DZ, Yan QY: **A comparative study of Jieyuanshen soup combined with citalopram in the treatment of depression [Chinese].** *Li Shizhen Medicine and Materia Medica Research* 2010, **21**: 2078-2079.

[2] Guo ZY: **Impact of paroxetine on post-stroke depression and activity of daily living [Chinese].** *Chinese Journal of Practical Nervous Diseases* 2008, 11: 112-113.

[3] Huang YP, Yuan G, Zhang J: **A comparative Study on Depression Treatment with a Combination of Fluoxetine and Amitriptyline [Chinese].** *Health Psychology Journal* 2003, **11**: 123-124.

[4] Kong M, Ding QE, Wang Y: **Comparative study of Paroxetine combine with Amitriptyline in the treatment of Depression and Anxiety [Chinese].** *China Prac Med* 2010, **5**: 1-2.

[5] Li F, Zhang Y, Ma L: **Observation of effect of early treatment of post-stroke depression [Chinese].** *Chin J Misdiagn* 2010, **10**: 2371-2372.

[6] Li QY, Wang XH, Liu J, Cheng J, Li XN, Zhang QJ, Wang YD, Gu JC, Li XZ, Zhang SR: **Control Study on Combined Treatment of Chinese Medicine and Western Medicine on Depression [Chinese].** *Chinese Archives of Traditional Chinese Medicine* 2009, **27**:1889-1891.

[7] Li XL, Zheng SY, Lu XB: **A clinical observation of the effect of Citaprolan with small-dose Quetiapine in depression [Chinese].** *Hainan Medical Journal* 2007, **18**: 30-32.

[8] Li Y, Zhang CF, Li H, Du W: **Observation of efficacy of fluoxetine in the treatment of post-stroke depression and neurologic deficit [Chinese].** *Clinical medicine and nursing research* 2009, **8**: 36-37.

[9] Liang XM: **Observation of clinical effect of fluoxetine in the treatment of depression [Chinese].** *China Medical Harald* 2010, **7**: 117.

[10] Liu EF: **Citalopram combined with amitriptyline in the treatment of post-stroke depressive disorder [Chinese].** *Journal of Shandong Medical College* 2006, **28**: 222-223.

[11] Liu XW, Li Q: **A comparative study of fluoxetine combined with clonazepam in the treatment of depression [Chinese].** *J Clin Psychol Med* 2001, **11**: 169-170.

[12] Mao ZC: **Clinical observation of fluoxetine in the treatment of post-stroke depression [Chinese].** *Clinical Education of General Practice* 2008, **6**: 60-61.

[13] Qian JJ, He BF, Shi YY: **Application of Risperidone in Depression without Psychotic Symptoms [Chinese].** *Chin J Rehabil Theory Pract* 2007, **13**: 477-478.

[14] Qian JJ, He BF, Shi YY, Gao S, Jin HL: **Comparison of risperidone combined with fluoxetine in treatment of depression without psychotic symptoms [Chinese].** *Chinese Journal of Clinical Pharmacy* 2008, **17**: 284-286.

[15] Qu W, Qin YY: **Improvement of sleep and anxiety in patients of major depression with fluoxetine combined with small dose of olanzapine [Chinese].** *Chinese Journal of Clinical Rehabilitation* 2005, **9**: 254-256.

[16] Ren QT, Wang TL: **A comparative observation of the efficacy between fluoxetine combined with clonazepam and fluoxetine combined with placebo in treatment depression [Chinese].** *Sichuan Mental Health* 2003, **16**: 94-95.

[17] Ren QT, Tian Y, Gao LH: **A comparative study of paroxetine combined with clonazepam in the treatment of depression [Chinese].** *Chin J Nerv Ment Dis* 2003, **29**: 70-71.

[18] Sun QX, Zeng DZ, Wang BH: **Efficacy and Safety of Citalopram Combined with Buspirone in Treatment of Patients with Post-stroke Depression [Chinese].** *Chinese Journal of Rehabilitation* 2007, **22**: 108-109.

[19] Sun YQ, Cheng FL, Zhao XY: **The efficacy and life quality comparison of typical and atypical antidepresant for depression [Chinese].** *Chinese Journal of Behavioral Medical Science* 2004, **13**: 530-531.

[20] Wang RF, Wang WZ: **Impact of fluoxetine on general rehabilitation of patients with post-stroke depression [Chinese].** *Chinese Journal of Practical Nervous Diseases* 2009, **12**: 54-56.

[21] Wang WY: **A comparative study of mirtazapine and citalopram in the treatment of senile depression [Chinese].** *Medical Journal of Chinese People's Health* 2009, **21**: 2981-2983.

[22] Wang XK, Lin Y, Zhang XL: **A comparative study of fluoxetine combined with buspirone in the treatment of depression [Chinese].** *Strait Pharmaceutical Journal* 2003, **15**: 46-47.

[23] Yang BQ, Zhang Z, Lin X, Ling AX, Wang LH, Zhao JF, Yang M: **Impact of cognitive behavior intervention combined with danhong injection and fluoxetine in the treatment of senile post-stroke depression and nerve function deficit rehabilization [Chinese].** *Zhejiang Journal of Traditional Chinese Medicine* 2010, **45**: 592-593.

[24] Zeng DZ, Hua SG, Fan XW: **Clinical study of citalopram combined with buspirone in treatment for senile depression [Chinese].** *Pract Geriatr* 2007, **21**: 194-196.

[25] Zhang GJ, Shi ZY, Liu S, Gong SH, Liu JS: **Clinical Observation on Treatment of Depression by Electro-Acupuncture Combined with Paroxetine [Chinese].** *Chin J Integr Med* 2007, **13**: 228-230.

[26] Zhao CL, Tan HX: **A comparative oberservation of fluoxetine combined with clonazepam in the treatment of depression [Chinese].** *Chinese Journal of Misdiagnostics* 2002, **2**: 1535.

[27] Zhao CL, Zhang Q: **A double-blind comparative analysis of paroxetine combined with clonazepam in the treatment of depression [Chinese].** *Chinese Journal of Clinical Rehabilitation* 2002, **6**: 1629.

[28] Zhao XW, Ren K, Jiang XY: **Observation of efficacy of seroxat combined with low-dose Risperdal in the treatment of treatment resistant depression [Chinese].** *Nervous Diseases and Mental Hygiene* 2004, **4**: 112.

**Exclusion Reason 4. not CCMD/ICD/DSM**

[1] Bi CX, Lin SH, Jiang L: **A comparative study of different therapeutic effectiveness in the treatment of post-stroke depression condition [Chinese].** *China Prac* *Med* 2010, **5**: 71-73.

[2] Gao YH: **Rehabilitating treatment of post-stroke depression [Chinese].** *China Medical Herald* 2009, 6: 45-46.

[3] Guo RY, Su L, Liu LA, Wang CX: **Effects of linggui bafa on the trerapeutic effect and quality of life in patients of post-stroke depression [Chinese].** *Chinese Acupuncture & Moxibustion* 2009, **29**: 785-790.

[4] Hu ZF, Feng Z: **Clinical observation of neurostan in the treatment of depression [Chinese].** *Journal of Shandong University of TCM* 2003, **27**: 45-46.

[5] Jiang XZ, Luo HC, Zhao XY: **Clinical research improvement of electric acupuncture treatment of depression [Chinese].** *Medical Journal of Chinese People Health* 2004, **16**: 36-38.

[6] Sun CY, Zhang JX: **Fluoxetine in the treatment of ischemic post-stroke depression [Chinese].** *Chinese Journal of Behavioral Medical Science* 2003, **12**: 398.

[7] Wang XQ, Zhang HY, Shu L, Du B, Jiao FY, Han ZC, Gao CG, Ai CS, Li LZ, Huang L: **Efficacy and safety of morinda officinalis oligose capsule in the treatment of mild or moderate depression [Chinese].** *Chinese Journal of New Drugs* 2009, **18**: 802-805.

[8] Xiao W, Kong HB, Wang Z, Wang Y, Wang J, Zeng YL, Zhu CQ, Guo T: **A clinical study of Qiangcongci combined with fluoxetine in the treatment of post-stroke depression [Chinese].** *CJTCM* 2009, **21**: 330-331.

[9] Yang XG, Lin YJ, Chen XD: **A clinical study of improvement of fluoxetine on post-stroke depression and nerve function deficit [Chinese].** *The Journal of Practical Medicine* 2009, **25**: 1127-1128.

[10] Zhang JQ, Li RW, Gu HW, Luo Z, Zhou BB: **Clinical observation of citalopram combined with risperdal in the treatment of post-schizophrenia depression [Chinese].** *Zhejiang medical Journal* 2008, **30**: 282-283.

[11] Zhao M, Wang ZM, Wang X, Ma JD: **The therapeutic observation of fluoxetine single or combined with psychotherapy in the depression succeeding brain stroke [Chinese].** *China Journal of Health Psychology* 1999, **7**: 241-243.

[12] Zhu JZ, Zhou ZX, Li ZJ: **Comparative study of citalopram vs mirtazapine in treatment of post-stroke depression [Chinese].** *Chinese Journal of Rehabilitation* 2009, **24**: 118-119.

[13] Zhu LP: **Observation of effect of Yangxinjieyu soup in the treatment of post-natal depression [Chinese].** *Chinese Primary Health Care* 2008, **22**: 86-87.

[14] Zhu Q, Duan XL**: A clinical observation of anshenjieyutang in the treatment of post-stroke depression [Chinese]. China Modern Doctor 2010, 48: 53-54.**

**Exclusion Reason 5. no relevant control group**

[1] Bai Y, Jiang HY, Xu XF: **The Phase II Clinical Trials for Escitalopram Oxalate to Treat Major Depressive Disorder [Chinese].** *Journal of Kunming Medical University* 2010, **12**: 22-26.

[2] Jin YJ, Chen ZJ: **Comparative Study on Sertraline (Made in China) and Zoloft in the Treatment of Depression [Chinese].** *Chinese General Practice* 2009, **9**: 495-496.

[3] Wang LH, Li MX, Wang CH, Shi YH, Ma ZW: **Comparison of efficacy between domestic and imported fluoxetine on depression [Chinese].** *Chinese Journal of Clinical Pharmacy* 2006, **15**: 307-309.

[4] Wang XQ, Zhou DF: **A comparative study of domestic and imported products of paroxetine on efficacy and safety in patients with major depression [Chinese].** *Shanghai Archives of Psychiatry* 2005, **17**: 334-336.

[5] Wu SJ, Wang LH: **Effect and Safety of Domestic and Imported Paroxetine in Treatment of Depression [Chinese].** *Occup* and Health 2009, 25: 106-107.

**Exclusion Reason 6. not RCT**

[1] Chen QX, Zhou HW: **Clinical observation of shuangliuwan in the treatment of post-stroke depression [Chinese].** *Journal of Changchun University of Traditional Chinese Medicine* 2007, **23**: 41-42.

[2] Sun HJ, Zhang XY: **Clinical analysis of paroxetine in the treatment of senile depression [Chinese].** *Journal of Inner Mongolia University for Nationalities* 2007, **13**: 1.

[3] Wu SY, Zhai M, Ding L, Zhang YZ, Zhu ZZ: **Analysis of effect of paroxetine in the treatment of depression [Chinese].** *J Clin Psychol Med* 1999, **9**: 112.

[4] Yao HX, Li GH: **Fluoxetine in the treatment of depression [Chinese].** *New Drugs and Clinical Remedies* 1997, **16**: 25-26.

**Exclusion Reason 7. not adult**

[1] Lee P, Shu L, Xu XF, Wang CY, Lee MS, Liu CY, Hong JP, Ruschel S, Raskin J, Colman SA, Harrison GA: **Once-daily duloxetine 60 mg in the treatment of major depressive disorder: Multicenter, double-blind, randomized, paroxetine-controlled, non-inferiority trial in China, Korea, Taiwan and Brazil.** *Psychiatry and Clinical Neurosciences* 2007, **61**: 295-307.

[2] Li CY, Zhou J: **Analysis of effect of fluoxetine in the treatment of 58 cases with depression [Chinese].** *Acta Academiae Medicinae Suzhou* 1997, **17**: 1149-1150.

[3] Zhao FY, Wu C: **76 cases of traditional Chinese medicine combined with western medicine in the treatment of depression [Chinese].** *Liaoning Journal of Traditional Chinese Medicine* 2010, **37**: 312-313.

**Exclusion Reason 8. no data**

[1] Guan NH, Zhang JP, Han ZL, Tang JX, Wei QL, Zhang WL, Jiao JJ, Ye HB, Wang XX: **A study on quality of life and cost of treatment for depression with sertraline, fluoxetine and paroxetine [Chinese].** *Chinese Journal of Behavioral Medical Science* 2002, **11**: 637-638.

[2] Li HY, Zhou DF, Song YQ, Fan JH, Luo HC, Zhao XY: **Effect on platelet protein kinase C of electro-acupuncture and fluoxetine treatment in patients with major depressive disorder [Chinese].** *Chinese Mental Health Journal* 2004, **18**: 688-691.

[3] Song YQ, Zhou DF, Fan JH, Luo HC, Zhao XY: **Effects of fluoxetine and electroacupuncture on G-protein level in platelet membrane from patients with major depression [Chinese].** *Chinese Mental Health Journal* 2004, **18**: 783-786.

**Exclusion Reason 9. Crossover**

[1] Fang YR, Yuan C, Xu Y, Chen J, Wu Z, Cao L, Yi ZH, Hong W, Wang Y, Jiang KD, Gao K, Cui XJ, Nierenberg AA, Operation study team: **Comparisons of the Efficacy and Tolerability of Extended-Release Venlafaxine, Mirtazapine, and Paroxetine in Treatment-Resistant Depression.** **A Double-Blind, Randomized Pilot Study in a Chinese Population.** *Journal of Clinical Psychopharmacology* 2010, **30**: 357-364.

**Exclusion Reason 10. Duplicate**

[1] Shu DH, Zhang K, He H, Han P: **A comparative study of paroxetine and amitriptyline in treatment of gerontism depression [Chinese].** *J Clin Psychol Med* 2005, **15**:64.

Appendix 3: Characteristics of included studies

Cai JY 2007

| Methods | 6 week double blind RCT |
| --- | --- |
| Participants | In- and outpatients  Inclusion criteria:  Depression according to CCMD-3  HAMD-17 ≥18, Age: /  Baseline Values:  HAMD score 22.4 +3.2 (reboxetine), 22.6+3.6 (citalopram)  Mean age: 32 years (reboxetine), 31 years (citalopram)  Women: 47% (reboxetine), 45% (citalopram) |
| Interventions | Reboxetine 4-8 mg/d: N=32  Citalopram 10-20 mg/d: N=31 |
| Outcomes | HAMD, TESS |
| Informed consent | Not reported |
| Financial support | Not reported |
| CONSORT 2010 | 35% items fulfilled |

Cao HJ 2008

| Methods | 6 week double blind RCT |
| --- | --- |
| Participants | Inpatients  Inclusion criteria:  Depression according to CCMD-3, Age: 18-55 years  HAMD-17 >18  Baseline Values:  HAMD score 22.86 +3.55 (bupropion), 22.71+3.67 (fluoxetine)  Age: 18-55 years  Women: 60% (bupropion), 58% (fluoxetine) |
| Interventions | Bupropion 300 mg/d: N=40  Fluoxetine 20 mg/d: N=40 |
| Outcomes | HAMD, MAMA, CGI, TESS |
| Informed consent | Not reported |
| Financial support | Not reported |
| CONSORT 2010 | 32% items fulfilled |

Chang SH 2006

| Methods | 6 week double blind RCT |
| --- | --- |
| Participants | In- and outpatients  Inclusion criteria:  Depression according to CCMD-3, Age: 60-70 years  HAMD≥24  Baseline Values:  HAMD score 27.9 +6.48 (sertraline), 28.61+6.73 (maprotiline)  Mean age: 67 years (sertraline), 68 years (maprotiline)  Women: 42% (sertraline), 45% (maprotiline) |
| Interventions | Sertraline 50-150 mg/d: N=31  Maprotiline 75-200 mg/d: N=31 |
| Outcomes | HAMD, TESS |
| Informed consent | Not reported |
| Financial support | Not reported |
| CONSORT 2010 | 32% items fulfilled |

Chen EM 2010

| Methods | 8 week double blind RCT |
| --- | --- |
| Participants | Inpatients  Inclusion criteria:  Depression according to CCMD-3  HAMD-17 ≥18, Age: 18-60 years  Baseline Values:  HAMD score 33.6 +4.2 (venlafaxine ER), 32.3+4.6 (paroxetine)  Mean age: 33 years (venlafaxine ER), 34 years (paroxetine)  Women: 88% (venlafaxine ER), 85% (paroxetine) |
| Interventions | Venlafaxine ER 75-225 mg/d: N=40  Paroxetine 20-60 mg/d: N=40 |
| Outcomes | HAMD, TESS |
| Informed consent | Not reported |
| Financial support | Not reported |
| CONSORT 2010 | 41% items fulfilled |

Chen LQ 2005

| Methods | 6 week double blind, double dummy RCT |
| --- | --- |
| Participants | In- and outpatients  Inclusion criteria:  Depression according to CCMD-3 or DSM-IV  HAMD ≥16, Age:  Baseline Values:  HAMD score 27.7 +6.23 (TCM), 27.1+5.65 (paroxetine)  Mean age: 37 years (TCM), 34 years (paroxetine)  Women: 36% (TCM), 37% (paroxetine) |
| Interventions | TCM 12 units/d: N=110  Paroxetine 20-40 mg/d: N=110 |
| Outcomes | HAMD, HAMA, CGI, TESS |
| Informed consent | Not reported |
| Financial support | Not reported |
| CONSORT 2010 | 49% items fulfilled |

Chen YH 2010

| Methods | 8 week double blind RCT |
| --- | --- |
| Participants | Inpatients  Inclusion criteria:  Depressive episode according to CCMD-3  HAMD-17 ≥18, Age: 60-75 years  Baseline Values:  HAMD score 26.6 +5.8 (escitalopram), 25.9+5.3 (mianserin)  Mean age: 63 years (escitalopram), 63 years (mianserin)  Women: 36% (escitalopram), 37% (mianserin) |
| Interventions | Escitalopram 5-15 mg/d: N=46  Mianserin 15-60 mg/d: N=46 |
| Outcomes | HAMD, TESS |
| Informed consent | Not reported |
| Financial support | Not reported |
| CONSORT 2010 | 30% items fulfilled |

Du B 2007

| Methods | 6 week double blind, double-dummy RCT |
| --- | --- |
| Participants | In- and outpatients  Inclusion criteria:  Depressive episode according to CCMD-3  HAMD-17 ≥17 and ≤28, Age: 18-65 years  Baseline Values:  HAMD score 20.49 +2.6 (TCM), 20.5+2.28 (fluoxetine)  Age: /  Women: / |
| Interventions | TCM 1440 mg/d: N=360  Fluoxetine 20 mg/d: N=120 |
| Outcomes | HAMD, HAMA, CGI |
| Informed consent | reported |
| Financial support | Not reported |
| CONSORT 2010 | 51% items fulfilled |

Du XS 2007

| Methods | 6 week double blind RCT |
| --- | --- |
| Participants | Inpatients  Inclusion criteria:  Depression according to CCMD-3  HAMD-17 >18, Age: 18-60 years  Baseline Values:  HAMD score 30.1 +4 (citalopram), 29.4+4.1 (fluoxetine)  Mean age: 35 years (citalopram), 34 years (fluoxetine)  Women: 44% (citalopram), 39% (fluoxetine) |
| Interventions | Citalopram 20 mg/d: N=36  Fluoxetine 20 mg/d: N=36 |
| Outcomes | HAMD, CGI-SI, TESS |
| Informed consent | Not reported |
| Financial support | Not reported |
| CONSORT 2010 | 43% items fulfilled |

Du XS 2009

| Methods | 8 week double blind RCT |
| --- | --- |
| Participants | Inpatients  Inclusion criteria:  Depression according to CCMD-3  HAMD>18, Age: >65 years  Baseline Values:  HAMD score 27.6 +5.6 (citalopram), 26.1+5.4 (sertraline)  Age: /  Women: 29% (citalopram), 36% (sertraline) |
| Interventions | Citalopram 20-40 mg/d: N=28  Sertraline 50-100 mg/d: N=28 |
| Outcomes | HAMD, TESS |
| Informed consent | Not reported |
| Financial support | Not reported |
| CONSORT 2010 | 38% items fulfilled |

Du YM 2006

| Methods | 6 week double blind RCT |
| --- | --- |
| Participants | Inpatients  Inclusion criteria:  Depression according to CCMD-3  HAMD-17≥18, Age: 20-65 years  Baseline Values:  HAMD score 26.9 +4.7 (fluoxetine), 26.8+5.2 (amitriptyline)  Mean age: 40 years (fluoxetine), 36 years (amitriptyline)  Women: 50% (fluoxetine), 50% (amitriptyline) |
| Interventions | Fluoxetine 20-40 mg/d: N=34  Amitriptyline 50-250 mg/d: N=34 |
| Outcomes | HAMD, CGI-SI, TESS |
| Informed consent | Not reported |
| Financial support | Not reported |
| CONSORT 2010 | 30% items fulfilled |

Fan HT 2007

| Methods | 6 week double blind RCT |
| --- | --- |
| Participants | In- and outpatients  Inclusion criteria:  Post-stroke Depression according to CCMD-3  HAMD-17≥18, Age: /  Baseline Values:  HAMD score 25.38 +5.25 (fluvoxamine), 26 +5.63 (sertraline)  Mean age: 55 years (fluvoxamine), 55 years (sertraline)  Women: 43% (fluvoxamine), 48% (sertraline) |
| Interventions | Fluvoxamine N=46  Sertraline N=46 |
| Outcomes | HAMD, MESSS, ADL |
| Informed consent | reported |
| Financial support | Not reported |
| CONSORT 2010 | 16% items fulfilled |

Fang LQ 2007

| Methods | 6 week double blind RCT |
| --- | --- |
| Participants | In- and outpatients  Inclusion criteria:  Post-stroke Depression, Depressive episode according to DSM-IV  HAMD-17≥18, Age: /  Baseline Values:  HAMD score: /  Mean age: 64 years (citalopram), 62 years (fluoxetine)  Women: 35% (citalopram), 40% (fluoxetine) |
| Interventions | Citalopram 10-40 mg/d: N=20  Fluoxetine 20-40 mg/d: N=20 |
| Outcomes | HAMD, CGI-SI, TESS |
| Informed consent | Not reported |
| Financial support | Not reported |
| CONSORT 2010 | 38% items fulfilled |

Gao YL 2006

| Methods | 6 week double blind RCT |
| --- | --- |
| Participants | In- and outpatients  Inclusion criteria:  Post-stroke Depression, Depression according to CCMD-3  Age: /  Baseline Values:  HAMD score: 27.98 +4.8 (fluoxetine), 28.12 +4.5 (amitriptyline)  Mean age: 59 years (fluoxetine), 58 years (amitriptyline)  Women: 51% (fluoxetine), 47% (amitriptyline) |
| Interventions | Fluoxetine 20 mg/d: N=37  Amitriptyline 50-175 mg/d: N=38 |
| Outcomes | HAMD, CGI-SI, TESS |
| Informed consent | Not reported |
| Financial support | Not reported |
| CONSORT 2010 | 35% items fulfilled |

Han GL 2006

| Methods | 6-8 week double blind RCT |
| --- | --- |
| Participants | In- and outpatients  Inclusion criteria:  Depressive episode according to CCMD-3  HAMD>18, Age: >60 years  Baseline Values:  HAMD score: /  Mean age: 65 years (amitriptyline), 62 years (fluoxetine)  Women: 58% (amitriptyline), 60% (fluoxetine) |
| Interventions | Amitriptyline 50-200 mg/d: N=30  Fluoxetine 20-50 mg/d: N=30 |
| Outcomes | HAMD, TESS |
| Informed consent | Not reported |
| Financial support | reported |
| CONSORT 2010 | 41% items fulfilled |
| Notes | the third arm Fluoxetine+TCM was not extracted |

Han ZL 2002

| Methods | 2 week double blind RCT |
| --- | --- |
| Participants | Inpatients  Inclusion criteria:  Depression according to CCMD-2-R  Age: /  Baseline Values:  HAMD score: 23.7+12.8 (sertraline), 25.5+14.2 (fluoxetine), 24+14.4 (paroxetine)  Age: 18-68 years, mean age 37.5 years  Women: 63% |
| Interventions | Sertraline 59 + 13 mg/d: N=26  Fluoxetine 23.6 + 6.4 mg/d: N=23  Paroxetine 22.2 + 4.4 mg/d: N=22 |
| Outcomes | HAMD, HAMA, TESS |
| Informed consent | Not reported |
| Financial support | Not reported |
| CONSORT 2010 | 35% items fulfilled |
| Notes | N_randomized_ not reported |

Hong CJ 2003

| Methods | 6 week double blind RCT |
| --- | --- |
| Participants | Outpatients  Inclusion criteria:  Major Depression according to DSM-IV,  HAMD-17≥15, Age: 18-75 years  Baseline Values:  HAMD score: 23.1+5.1 (mirtazapine), 24.3+5.2 (fluoxetine)  Mean age: 47 years (mirtazapine), 47 (fluoxetine)  Women: 62% (mirtazapine), 64% (fluoxetine) |
| Interventions | Mirtazapine 15-45 mg/d: N=66  Fluoxetine 20-40 mg/d: N=66 |
| Outcomes | HAMD, CGI |
| Informed consent | reported |
| Financial support | reported |
| CONSORT 2010 | 62% items fulfilled |
| Notes | N_randomized_ not reported for each group, English |

Hsu JW 2011

| Methods | 6 week double blind, double dummy RCT |
| --- | --- |
| Participants | Outpatients  Inclusion criteria:  Major Depression according to DSM-IV  MADRS≥25, Age: 20-65 years  Baseline Values:  MADRS score: 36.6+5.5 (citalopram), 38.2+4.9 (sertraline)  Mean age: 43 years (citalopram), 38 (sertraline)  Women: 52% (citalopram), 67% (sertraline) |
| Interventions | Citalopram 20 mg/d: N=25  Sertraline 50 mg/d: N=26 |
| Outcomes | MADRS |
| Informed consent | reported |
| Financial support | reported |
| CONSORT 2010 | 59% items fulfilled |
| Notes | English |

Huang P 2006

| Methods | 6 week double blind RCT |
| --- | --- |
| Participants | In- and outpatients  Inclusion criteria:  Post-stroke Depression, Depression according to CCMD-3  HAMD-17≥18, Age: /Baseline Values:  HAMD score: 25.38+5.25 (citalopram), 26.05+5.63 (fluoxetine)  Mean age: 55 years (citalopram), 57 years (fluoxetine)  Women: 43% (citalopram), 48% (fluoxetine) |
| Interventions | Citalopram 20-40 mg/d: N=46  Fluoxetine 20-40 mg/d: N=46 |
| Outcomes | HAMD, MESSS, ADL, TESS |
| Informed consent | reported |
| Financial support | Not reported |
| CONSORT 2010 | 46% items fulfilled |

Hu MR 2009

| Methods | 6 week double blind RCT |
| --- | --- |
| Participants | Outpatients  Inclusion criteria:  Depressive episode according to CCMD-3  HAMD-17≥18, Age: 18-60 years  Baseline Values:  HAMD score: 21.36+2.69 (escitalopram), 20.78+2.43 (citalopram)  Mean age: 31 years (escitalopram), 31 years (citalopram)  Women: 52% (escitalopram), 57% (citalopram) |
| Interventions | Escitalopram 10-20 mg/d: N=25  Citalopram 20-40 mg/d: N=23 |
| Outcomes | HAMD, CGI |
| Informed consent | reported |
| Financial support | Not reported |
| CONSORT 2010 | 35% items fulfilled |

Jiang T 2010

| Methods | 6 week double blind, double-dummy RCT |
| --- | --- |
| Participants | In- and outpatients  Inclusion criteria:  Depressive episode according to CCMD-3  HAMD-17≥18, Age: 18-60 years  Baseline Values:  HAMD score: 24+4 (nefazotone), 24+5 (fluoxetine)  Age: 18-60 years (nefazotone), 18-60 years (fluoxetine)  Women: 38% (nefazotone), 40% (fluoxetine) |
| Interventions | Nefazotone 300-500 mg/d: N=120  Fluoxetine 20-40 mg/d: N=120 |
| Outcomes | HAMD, HAMA, CGI, TESS |
| Informed consent | reported |
| Financial support | Not reported |
| CONSORT 2010 | 51% items fulfilled |

Jiang XY 2009

| Methods | 6 week double blind RCT |
| --- | --- |
| Participants | In- and outpatients  Inclusion criteria:  Depressive episode according to CCMD-3  HAMD-17≥18, Age: 18-60 years  Baseline Values:  HAMD score: 23.82+2.53 (escitalopram), 23.46+2.06 (citalopram)  Mean age: 39 years (escitalopram), 39 years (citalopram)  Women: 56% (escitalopram), 53% (citalopram) |
| Interventions | Escitalopram 5-20 mg/d: N=32  Citalopram 20-60 mg/d: N=32 |
| Outcomes | HAMD, HAMA, TESS |
| Informed consent | Not reported |
| Financial support | Not reported |
| CONSORT 2010 | 46% items fulfilled |

Kong QM 2011

| Methods | 6 week double blind, double dummy RCT |
| --- | --- |
| Participants | In- and outpatients  Inclusion criteria:  Depressive episode according to ICD-10  HAMD-17≥18, Age: 18-65 years  Baseline Values:  HAMD score: 22.48+2.75 (Morinda officinalisoligose), 22.53+2.52 (Fluoxetine), 22.35+2.97 (Placebo)  Mean age: 40 years (Morinda officinalisoligose), 37 years (Fluoxetine), 38 years (Placebo)  Women: 57% (Morinda officinalisoligose), 57% (Fluoxetine), 59% (Placebo) |
| Interventions | Morinda officinalisoligose 600 mg/d: N=367  Fluoxetine 30 mg/d: N=123  Placebo Morinda officinalisoligose 300 mg/d: N=123 |
| Outcomes | HAMD, HAMA, CGI |
| Informed consent | reported |
| Financial support | Not reported |
| CONSORT 2010 | 49% items fulfilled |

Kong YB 2004

| Methods | 8 week double blind RCT |
| --- | --- |
| Participants | In- and outpatients  Inclusion criteria:  Post-stroke Depression, Depression according to CCMD-3  HAMD-17≥18, Age: /  Baseline Values:  HAMD score: /  Mean age: 63 years (citalopram), 62 years (amitriptyline)  Women: 38% (citalopram), 43% (amitriptyline) |
| Interventions | Citalopram 10-40 mg/d: N=21  Amitriptyline 25-150 mg/d: N=21 |
| Outcomes | HAMD, CGI-SI, TESS |
| Informed consent | Not reported |
| Financial support | Not reported |
| CONSORT 2010 | 35% items fulfilled |

Li B 1996

| Methods | 6 week double blind RCT |
| --- | --- |
| Participants | Inpatients  Inclusion criteria:  Depression according to ICD-10  HAMD-17≥18, Age:18-65 years  Baseline Values:  HAMD score: 26.9+4.7 (sertraline), 26.8+5.2 (amitriptyline)  Mean age: 40 years (sertraline), 36 years (amitriptyline)  Women: 50% (sertraline), 49% (amitriptyline) |
| Interventions | Sertraline 50-200 mg/d: N=68  Amitriptyline 50-250 mg/d: N=68 |
| Outcomes | HAMD, CGI, TESS |
| Informed consent | Not reported |
| Financial support | Not reported |
| CONSORT 2010 | 43% items fulfilled |

Li GJ 2005

| Methods | 6 week double blind, double-dummy RCT |
| --- | --- |
| Participants | Outpatients  Inclusion criteria:  Depression according to CCMD-3  HAMD-17≥18, Age:18-65 years  Baseline Values:  HAMD score: 21.6+2.6 (bupropion), 22.5+4 (fluoxetine)  Mean age: 41 years (bupropion), 40 years (fluoxetine)  Women: 67% (bupropion), 63% (fluoxetine) |
| Interventions | Bupropion 150 mg/d: N=30  Fluoxetine 20 mg/d: N=30 |
| Outcomes | HAMD, CGI, TESS |
| Informed consent | reported |
| Financial support | Not reported |
| CONSORT 2010 | 51% items fulfilled |

Li HF 2006

| Methods | 6 week double blind, double-dummy RCT |
| --- | --- |
| Participants | Inclusion criteria:  Depressive episode according to CCMD-3  HAMD-17≥18, Age:18-65 years  Baseline Values:  HAMD score: 27.46+5.56 (bupropion), 26.8+5.46 (fluoxetine)  Mean age: 38 years (bupropion), 41 years (fluoxetine)  Women: 51% (bupropion), 53% (fluoxetine) |
| Interventions | Bupropion 300 mg/d: N=104  Fluoxetine 20 mg/d: N=104 |
| Outcomes | HAMD, HAMA, CGI, TESS |
| Informed consent | reported |
| Financial support | Not reported |
| CONSORT 2010 | 43% items fulfilled |

Li HF 2007

| Methods | 6 week double blind, double-dummy RCT |
| --- | --- |
| Participants | In- and outpatients  Inclusion criteria:  Depressive episode according to CCMD-3  HAMD-17≥18, HAMA≥14, Age:18-65 years  Baseline Values:  HAMD score: 26.28+4.64 (reboxetine), 26.16+4.86 (fluoxetine)  Mean age: 39 years (reboxetine), 38 years (fluoxetine)  Women: 55% (reboxetine), 63% (fluoxetine) |
| Interventions | Reboxetine 8 mg/d: N=67  Fluoxetine 20 mg/d: N=70 |
| Outcomes | HAMD, HAMA, TESS |
| Informed consent | reported |
| Financial support | Not reported |
| CONSORT 2010 | 43% items fulfilled |

Li HJ 2011

| Methods | 6 week double blind, double-dummy RCT |
| --- | --- |
| Participants | Inclusion criteria:  Post-stroke Depression, Depressive episode according to DSM-IV,  Age: >18 years  Baseline Values:  HAMD score: 24.8+5 (Acupuncture), 26.7+4.6 (fluoxetine)  Mean age: 57 years (Acupuncture), 59 years (fluoxetine)  Women: 48% (Acupuncture), 50% (fluoxetine) |
| Interventions | Acupuncture: N=23  Fluoxetine 10-30 mg/d: N=20 |
| Outcomes | HAMD, ASES |
| Informed consent | reported |
| Financial support | Not reported |
| CONSORT 2010 | 68% items fulfilled |

Li J 2006

| Methods | 6 week double blind RCT |
| --- | --- |
| Participants | Outpatients  Inclusion criteria:  Depression according to CCMD-3  HAMD-17≥18, Age: 18-65 years  Baseline Values:  HAMD score: 22.5+2.9 (escitalopram), 21.1+2.4 (citalopram)  Mean age: 37 years (escitalopram), 34 years (citalopram)  Women: 54% (escitalopram), 54% (citalopram) |
| Interventions | Escitalopram 10-20 mg/d: N=28  Citalopram 20-40 mg/d: N=28 |
| Outcomes | HAMD, CGI, HAMA |
| Informed consent | reported |
| Financial support | Not reported |
| CONSORT 2010 | 62% items fulfilled |

Li J 2007

| Methods | 8 week double blind RCT |
| --- | --- |
| Participants | Inpatients  Inclusion criteria:  Depressive episode according to ICD-10, refractory depression  HAMD-17≥20, Age: 18-60 years, Baseline Values:  HAMD score: 32.8+4.8 (mirtazapine), 32.8+4.8 (paroxetine)  Mean age: 34 years (mirtazapine), 33 years (paroxetine)  Women: 85% (mirtazapine), 83% (paroxetine) |
| Interventions | Mirtazapine 30 mg/d: N=40  Paroxetine 20 mg/d: N=40 |
| Outcomes | HAMD, TESS, QOL |
| Informed consent | reported |
| Financial support | Not reported |
| CONSORT 2010 | 41% items fulfilled |

Li LJ 2010

| Methods | 8 week double blind, double-dummy RCT |
| --- | --- |
| Participants | In- and outpatients  Inclusion criteria:  Depression according to CCMD-3  HAMD-24>18, Age: /  Baseline Values:  HAMD score: 22.36+5.82 (TCM), 23.38+5.64 (fluoxetine)  Age: 54-78 years (TCM), 58-72 years (fluoxetine)  Women: 53% (TCM), 57% (fluoxetine) |
| Interventions | TCM 4.05 g/d: N=30  Fluoxetine 20 mg/d: N=30 |
| Outcomes | HAMD, MMSE, CGI, NDS |
| Informed consent | Not reported |
| Financial support | reported |
| CONSORT 2010 | 49% items fulfilled |

Li N 2006

| Methods | 4 week double blind RCT |
| --- | --- |
| Participants | In- and outpatients  Inclusion criteria:  Depression according to CCMD-3  HAMD>14, Age: 55-70 years  Baseline Values:  HAMD score: 19.82+7.83 (reboxetine), 18.96+9.71 (fluoxetine)  Mean age: 64 years (reboxetine), 65 years (fluoxetine)  Women: 40% (reboxetine), 47% (fluoxetine) |
| Interventions | Reboxetine 4-8 mg/d: N=30  Fluoxetine 20-40 mg/d: N=30 |
| Outcomes | HAMD, HAMA, CGI, TESS |
| Informed consent | reported |
| Financial support | Not reported |
| CONSORT 2010 | 43% items fulfilled |

Li N 2007

| Methods | 6 week double blind RCT |
| --- | --- |
| Participants | In- and outpatients  Inclusion criteria:  Depression according to CCMD-3  HAMD-17≥18, Age: /  Baseline Values:  HAMD score: 22.4+3.9 (duloxetine), 23+4.3 (fluoxetine)  Mean age: 38 years (duloxetine), 37 years (fluoxetine)  Women: 56% (duloxetine), 59% (fluoxetine) |
| Interventions | Duloxetine 30-60 mg/d: N=34  Fluoxetine 10-20 mg/d: N=34 |
| Outcomes | HAMD, HAMA, CGI, AE |
| Informed consent | reported |
| Financial support | Not reported |
| CONSORT 2010 | 30% items fulfilled |

Li XX 2010

| Methods | 6 week double blind RCT |
| --- | --- |
| Participants | Inclusion criteria:  Depression according to CCMD-3  Age: 18-65 years  Baseline Values:  HAMD score: /  Age: 18-65  Women: / |
| Interventions | Escitalopram 10-20 mg/d: N=24  Citalopram 20-40 mg/d: N=24 |
| Outcomes | HAMD, CGI |
| Informed consent | reported |
| Financial support | Not reported |
| CONSORT 2010 | 43% items fulfilled |

Liu SS 2011

| Methods | 6 week double blind, double dummy RCT |
| --- | --- |
| Participants | Outpatients  Inclusion criteria:  Depression according to CCMD-3  HAMD-17≥18, Age: 18-65 years  Baseline Values:  HAMD score:  Mean age: 39 years (Kexinshu), 39 years (fluoxetine)  Women: 59% (kexinshu), 58% (fluoxetine) |
| Interventions | Kexinshu 4.5 g/d: N=341  Fluoxetine 10 mg/d: N=114 |
| Outcomes | HAMD |
| Informed consent | reported |
| Financial support | Not reported |
| CONSORT 2010 | 54% items fulfilled |

Lu XJ 2008

| Methods | 6 week double blind RCT |
| --- | --- |
| Participants | In- and outpatients  Inclusion criteria:  Depressive episode according to CCMD-3  HAMD-17≥18, Age: /  Baseline Values:  HAMD score: 27.4+4.1 (venlafaxine), 28.6+3.8 (paroxetine)  Mean age: 41 years (venlafaxine), 40 years (paroxetine)  Women: 55% (venlafaxine), 56% (paroxetine) |
| Interventions | Venlafaxine 50-250 mg/d: N=88  Paroxetine 20-40 mg/d: N=82 |
| Outcomes | HAMD, HAMA, TESS |
| Informed consent | Not reported |
| Financial support | Not reported |
| CONSORT 2010 | 41% items fulfilled |

Luo HC 2003

| Methods | 6 week double blind, double dummy RCT |
| --- | --- |
| Participants | Outpatients  Inclusion criteria:  Depression according to DSM-IV, ICD-10  HAMD≥20, Age: 18-65 years  Baseline Values:  HAMD score: 22.42+2.93 (TCM), 22.16+2.16 (fluoxetine), 22.84+3.47 (placebo)  Mean age:30 years (TCM), 34 (fluoxetine), 32 (placebo)  Women: 58% |
| Interventions | TCM: N=31  Fluoxetine 20 mg/d: N=32  Placebo: N=32 |
| Outcomes | HAMD, SERS, SDS, CGI |
| Informed consent | reported |
| Financial support | Not reported |
| CONSORT 2010 | 43% items fulfilled |

Lv ZL 2013

| Methods | 6 week double blind RCT |
| --- | --- |
| Participants | Outpatients  Inclusion criteria:  Depression according to CCMD-3  HAMD-17≥18, Age: ≥18 years  Baseline Values:  HAMD score: 23.06+2.22 (Escitalopram), 22.78+2.03 (citalopram)  Mean age:32 years (Escitalopram), 32 years (citalopram)  Women: 50% (escitalopram), 50% (citalopram) |
| Interventions | Escitalopram: 10-20 mg/d, N=20  Citalopram: 20-40 mg/d: N=22 |
| Outcomes | HAMD |
| Informed consent | reported |
| Financial support | Not reported |
| CONSORT 2010 | 46% items fulfilled |

Ma X 2007

| Methods | 6 week double blind RCT |
| --- | --- |
| Participants | In- and outpatients  Inclusion criteria:  Post-stroke Depression, Depression according to CCMD-3  HAMD-17≥17, Baseline Values:  HAMD score: 31.12+2.23 (TCM), 31.05+2.07 (fluoxetine)  Mean age: 57 years (TCM), / years (fluoxetine)  Women: 52% (TCM), / (fluoxetine) |
| Interventions | TCM: N=42  Fluoxetine 20 mg/d: N=35 |
| Outcomes | HAMD |
| Informed consent | Not reported |
| Financial support | Not reported |
| CONSORT 2010 | 30% items fulfilled |

Mao PX 2008

| Methods | 8 week double blind, double-dummy RCT |
| --- | --- |
| Participants | In- and outpatients  Inclusion criteria:  Depressive episode according to DSM-IV  HAMD-17≥18, CGI-S≥4, Age: 18-65 years  Baseline Values:  HAMD: 24.7+5.4 (escitalopram), 24.1+4.5 (fluoxetine)  Mean age: 37 years (escitalopram), 41 (fluoxetine)  Women: 50% (escitalopram), 63% (fluoxetine) |
| Interventions | Escitalopram 10 mg/d: N=123  Fluoxetine 20 mg/d: N=117 |
| Outcomes | HAMD, MADRS |
| Informed consent | Not reported |
| Financial support | Reported (by Pharmaceutical Company) |
| CONSORT 2010 | 73% items fulfilled |
| Notes | English and design in accordance with the regulations of the Chinese State Food and Drug Administration on clinical trial guidelines for imported drugs |

Mao PX 2010

| Methods | 6 week double blind RCT |
| --- | --- |
| Participants | In- and outpatients  Inclusion criteria:  Depressive episode according to DSM-IV  HAMD-17≥18, Age: 18-65 years  Baseline Values:  HAMD score: 23+3 (reboxetine), 22+3 (fluoxetine)  Mean age: 38 years (reboxetine), 39 years (fluoxetine)  Women: 54% (reboxetine), 64% (fluoxetine) |
| Interventions | Reboxetine 4-8 mg/d: N=120  Fluoxetine 10-20 mg/d: N=120 |
| Outcomes | HAMD, HAMA, CGI-I, CGI-S |
| Informed consent | reported |
| Financial support | Not reported |
| CONSORT 2010 | 54% items fulfilled |

Meng Y 2002

| Methods | 6 week double blind RCT |
| --- | --- |
| Participants | In- and outpatients  Inclusion criteria:  Depression according to CCMD-2-R  HAMD≥24, Age: ≥60 years  Baseline Values:  HAMD score: 26.8+4.8 (sertraline), 26.8+5 (amitriptyline)  Mean age: 68 years (sertraline), 70 years (amitriptyline)  Women: 42% (sertraline), 47% (amitriptyline) |
| Interventions | Sertraline 50-150 mg/d: N=19  Amitriptyline 50-200 mg/d: N=21 |
| Outcomes | HAMD, CGI, TESS |
| Informed consent | Not reported |
| Financial support | Not reported |
| CONSORT 2010 | 43% items fulfilled |

Ou HX 2001

| Methods | 6 week double blind, double dummy RCT |
| --- | --- |
| Participants | In- and outpatients  Inclusion criteria:  Depressive episode according to CCMD-2-R  HAMD-17≥18, Age: 18-64 years  Baseline Values:  HAMD score: 28.9+5.5 (venlafaxine), 28.4+4.6 (fluoxetine)  Age: 18-64 years  Women: / |
| Interventions | Venlafaxine: N=15  Fluoxetine: N=15 |
| Outcomes | HAMD, HAMA, CGI, TESS |
| Informed consent | Not reported |
| Financial support | Not reported |
| CONSORT 2010 | 35% items fulfilled |

Ou JJ 2011

| Methods | 6 week double blind RCT |
| --- | --- |
| Participants | In- and outpatients  Inclusion criteria:  MDD according to DSM-IV-TR, HAMD-17≥17, Age: 18-65 years  Baseline Values:  HAMD score: 23+4 (escitalopram), 22.9+4.4 (citalopram)  Mean age: 37 years (escitalopram), 36 years (citalopram)  Women: 58% (escitalopram), 54% (citalopram) |
| Interventions | Escitalopram: 10-20 mg/d, N=120  Citalopram: 20-40 mg/d, N=120 |
| Outcomes | HAMD, AEs |
| Informed consent | reported |
| Financial support | reported |
| CONSORT 2010 | 81% items fulfilled |
| Notes | English |

Peng YX 2007

| Methods | 8 week double blind RCT |
| --- | --- |
| Participants | Inpatients  Inclusion criteria:  Post-stroke Depression, Depressive episode according to CCMD-3  HAMD-17≥18, Age: /  Baseline Values:  HAMD score: /  Mean age: 64 years (fluoxetine), 64 (amitriptyline)  Women: 38% (fluoxetine), 43% (amitriptyline) |
| Interventions | Fluoxetine 10-40 mg/d: N=21  Amitriptyline 25-150 mg/d: N=21 |
| Outcomes | HAMD, CGI-SI, TESS |
| Informed consent | Not reported |
| Financial support | Not reported |
| CONSORT 2010 | 35% items fulfilled |

Qu M 2007

| Methods | 6 week double blind RCT |
| --- | --- |
| Participants | Outpatients  Inclusion criteria:  Depression according to CCMD-3  HAMD≤35, Age: ≤65 years  Baseline Values:  HAMD score: 24.52+3.95 (TCM), 21.35+6.76 (fluoxetine)  Age: 21-65 years, mean age 42 years  Women: 60% |
| Interventions | TCM: N=38  Fluoxetine 20 mg/d: N=35 |
| Outcomes | HAMD |
| Informed consent | Not reported |
| Financial support | reported |
| CONSORT 2010 | 49% items fulfilled |

Shi SX 1997

| Methods | 6 week double blind RCT |
| --- | --- |
| Participants | Inclusion criteria:  Depression according to CCMD-2, DSM-III-R  HAMD-17≥18, Age: 18-65 years  Baseline Values:  HAMD score: 27.43+5.32 (paroxetine), 28.23+5.49 (amitriptyline)  Mean age: 39 years (paroxetine), 42 years (amitriptyline)  Women: 41% (paroxetine), 30% (amitriptyline) |
| Interventions | Paroxetine 20-30 mg/d: N=32  Amitriptyline 175 mg/d: N=33 |
| Outcomes | HAMD, HAMA, CGI, TESS |
| Informed consent | Not reported |
| Financial support | Not reported |
| CONSORT 2010 | 41% items fulfilled |

Shu DH 2004

| Methods | 6 week double blind RCT |
| --- | --- |
| Participants | Inpatients  Inclusion criteria:  Depressive episode according to ICD-10  HAMD-21≥22, Age: >65 years  Baseline Values:  HAMD score: 31.3+4.3 (paroxetine), 30.7+3.9 (amitriptyline)  Mean age: 39 years (paroxetine), 42 years (amitriptyline)  Women: 41% (paroxetine), 30% (amitriptyline) |
| Interventions | Paroxetine 20 mg/d: N=19  Amitriptyline 50 mg/d: N=19 |
| Outcomes | HAMD, TESS |
| Informed consent | Not reported |
| Financial support | Not reported |
| CONSORT 2010 | 27% items fulfilled |

Sun SH 2001

| Methods | 6 week double blind RCT |
| --- | --- |
| Participants | Inpatients  Inclusion criteria:  Depression according to CCMD-2-R  HAMD-17≥18, Age: /  Baseline Values:  HAMD score: 28.82+7.01 (fluoxetine), 27.96+6.64 (doxepin)  Mean age: 37 years |
| Interventions | Fluoxetine 20-40 mg/d: N=30  Doxepin 50-150 mg/d: N=30 |
| Outcomes | HAMD, CGI, TESS |
| Informed consent | Not reported |
| Financial support | Not reported |
| CONSORT 2010 | 30% items fulfilled |

Sun XL 1997

| Methods | 6 week double blind RCT |
| --- | --- |
| Participants | Inpatients  Inclusion criteria:  Depression according to CCMD-2-R  HAMD≥17, Age: /Baseline Values:  HAMD score: 25.53+4.58 (paroxetine), 24+4.9 (sertraline), 25.13+5.27 (amitriptyline)  Mean age: 39 years (paroxetine), 32 (sertraline), 35 (amitriptyline)  Women: 29% (paroxetine), 29% (sertraline), 53% (amitriptyline) |
| Interventions | Paroxetine 20-30 mg/d: N=17  Sertraline 20-40 mg/d: N=17  Amitriptyline 140-210 mg/d: N=19 |
| Outcomes | HAMD, TESS |
| Informed consent | Not reported |
| Financial support | Not reported |
| CONSORT 2010 | 43% items fulfilled |

Tan XG 2004

| Methods | 6 week double blind RCT |
| --- | --- |
| Participants | Inpatients  Inclusion criteria: Depression according to CCMD-3,  HAMD-17>18, Age: >65 years  Baseline Values:  HAMD score: 30.5+2.1 (citalopram), 31.2+2.3 (amitriptyline)  Mean age: 68 years (citalopram), 69 (amitriptyline)  Women: 28% (citalopram), 23% (amitriptyline) |
| Interventions | Citalopram 20-40 mg/d: N=25  Amitriptyline 100-200 mg/d: N=26 |
| Outcomes | HAMD, TESS |
| Informed consent | Not reported |
| Financial support | Not reported |
| CONSORT 2010 | 35% items fulfilled |

Wang XQ 2009

| Methods | 6 week double blind RCT |
| --- | --- |
| Participants | In- and outpatients  Inclusion criteria:  Depressive episode according to ICD-10  HAMD-17>17 and ≤24, Age: 18-65 years  Baseline Values:  HAMD score: 20.9+1.9 (TCM high), 20.8+2 (TCM), 20.9+2 (fluoxetine)  Mean age: 40 y (TCM high), 40 (TCM), 40 (fluoxetine)  Women: 56% (TCM high), 61% (TCM), 53% (fluoxetine) |
| Interventions | TCM high 400 or 800 mg/d: N=119  TCM 300 or 600 mg/d: N=121  Fluoxetine 20-30 mg/d: N=121 |
| Outcomes | HAMD, CGI |
| Informed consent | Not reported |
| Financial support | Not reported |
| CONSORT 2010 | 49% items fulfilled |

Wei J 2008

| Methods | 8 week double blind RCT |
| --- | --- |
| Participants | In- and outpatients  Inclusion criteria:  Post-stroke Depression, Depression according to CCMD-3  HAMD-17≥18, Age: /  Baseline Values:  HAMD score: 28.4+3.1 (citalopram), 27.8+4.6 (amitriptyline)  Mean age: 63 years (citalopram), 62 years (amitriptyline)  Women: 38% (citalopram), 43% (amitriptyline) |
| Interventions | Citalopram 10-40 mg/d: N=21  Amitriptyline 50-250 mg/d: N=21 |
| Outcomes | HAMD, CGI-SI, TESS |
| Informed consent | Not reported |
| Financial support | Not reported |
| CONSORT 2010 | 35% items fulfilled |

Wu Y 2009

| Methods | 6 week double blind, double-dummy RCT |
| --- | --- |
| Participants | In- and outpatients  Inclusion criteria:  Depressive episode according to CCMD-3  HAMD-17≥18 and HAMA≥14, Age: 18-65 years  Baseline Values:  HAMD score: /  Mean age: /  Women: / |
| Interventions | Bupropion 300 mg/d: N=89  Fluoxetine 20 mg/d: N=80 |
| Outcomes | HAMD, HAMA, TESS |
| Informed consent | reported |
| Financial support | Not reported |
| CONSORT 2010 | 46% items fulfilled |

Xiang H 1998

| Methods | 6 week double blind RCT |
| --- | --- |
| Participants | Inclusion criteria:  Depressive episode according to CCMD-2-R  HAMD>18, Age: 18-65 years  Baseline Values:  HAMD score: 27.6+1.93 (paroxetine), 26.9+1.72 (amitriptyline)  Mean age: 37 years (paroxetine), 35 years (amitriptyline)  Women: 53% (paroxetine), 57% (amitriptyline) |
| Interventions | Paroxetine 20-40 mg/d: N=30  Amitriptyline 25-250 mg/d: N=30 |
| Outcomes | HAMD, CGI, TESS |
| Informed consent | Not reported |
| Financial support | Not reported |
| CONSORT 2010 | 32% items fulfilled |

Xiao JS 2005

| Methods | 8 week double blind RCT |
| --- | --- |
| Participants | Inpatients  Inclusion criteria:  Post-stroke Depression, Depressive episode according to CCMD-3, ICD-10  Age: /  Baseline Values:  HAMD score: 20.8+3.7 (TCM), 21.4+2.2 (fluoxetine)  Age: 22-80 years  Women: 33% |
| Interventions | TCM 1000-2000 mg/d: N=52  Fluoxetine 20-40 mg/d: N=50 |
| Outcomes | HAMD, TESS |
| Informed consent | Not reported |
| Financial support | Not reported |
| CONSORT 2010 | 32% items fulfilled |

Xie GR 1998

| Methods | 6 week double blind RCT |
| --- | --- |
| Participants | Inpatients  Inclusion criteria:  Depression according to CCMD-2-R, DSM-III-R  HAMD-21≥18, Age: 18-65 years  Baseline Values:  HAMD score: 27+5.24 (paroxetine), 26.96+5.16 (amitriptyline)  Mean age: 37 years (paroxetine), 38 years (amitriptyline)  Women: 39% (paroxetine), 50% (amitriptyline) |
| Interventions | Paroxetine 20-30 mg/d: N=44  Amitriptyline 25-175 mg/d: N=46 |
| Outcomes | HAMD, HAMA, CGI, TESS |
| Informed consent | reported |
| Financial support | Not reported |
| CONSORT 2010 | 41% items fulfilled |

Xie SY 2008

| Methods | 6 week double blind RCT |
| --- | --- |
| Participants | Inpatients  Inclusion criteria:  Depression according to CCMD-3, first episode depression  HAMD-17>18, , Age: 18-60 years  Baseline Values:  HAMD score: 28.7+6.4 (sertraline), 27.3+5.8 (fluoxetine)  Mean age: 35 years (sertraline), 33 years (fluoxetine)  Women: 44% (sertraline), 39% (fluoxetine) |
| Interventions | Sertraline 50 mg/d: N=36  Fluoxetine 20 mg/d: N=36 |
| Outcomes | HAMD, CGI-SI, TESS |
| Informed consent | Not reported |
| Financial support | Not reported |
| CONSORT 2010 | 49% items fulfilled |

Xu YC 1998

| Methods | 6 week double blind RCT |
| --- | --- |
| Participants | Inclusion criteria:  Depression according to CCMD-2, DSM-III-R  HAMD-17≥18, Age: 18-65 years  Baseline Values:  HAMD score: 27.43+5.32 (sertraline), 28.23+5.49 (amitriptyline)  Mean age: /  Women: / |
| Interventions | Sertraline 50-200 mg/d: N=32  Amitriptyline 50-250 mg/d: N=33 |
| Outcomes | HAMD, CGI, TESS |
| Informed consent | Not reported |
| Financial support | Not reported |
| CONSORT 2010 | 32% items fulfilled |

Xun GL 2009

| Methods | 6 week double blind, double-dummy RCT |
| --- | --- |
| Participants | In- and outpatients  Inclusion criteria:  Depressive episode according to CCMD-3  HAMD-17≥17, Age: 18-65 years  Baseline Values:  HAMD score: 23+4 (escitalopram), 23+4 (citalopram)  Mean age: 37 years (escitalopram), 36 years (citalopram)  Women: 56% (escitalopram), 53% (citalopram) |
| Interventions | Escitalopram 10-20 mg/d: N=120  Citalopram 20-40 mg/d: N=120 |
| Outcomes | HAMD, CGI |
| Informed consent | reported |
| Financial support | Not reported |
| CONSORT 2010 | 51% items fulfilled |

You NX 2000

| Methods | 4 week double blind RCT |
| --- | --- |
| Participants | Inclusion criteria:  Depression according to CCMD-II-R  HAMD≥17, SDS≥53, Age: /  Baseline Values:  HAMD score: 25.5+7.4 (citalopram), 24.2+2.3 (fluoxetine)  Age: 20-60 years  Women: 69% |
| Interventions | Citalopram 20 mg/d: N=36  Fluoxetine 20 mg/d: N=36 |
| Outcomes | HAMD, SDS |
| Informed consent | Not reported |
| Financial support | Not reported |
| CONSORT 2010 | 22% items fulfilled |

Yu MH 1996

| Methods | 6 week double blind RCT |
| --- | --- |
| Participants | Inpatients  Inclusion criteria:  Depression according to CCMD-2-R, DSM-III-R  HAMD-17>20, Age: ≥55 years  Baseline Values:  HAMD score: 39.22+2.86 (fluoxetine), 38.81+4.29 (amitriptyline)  Mean age: 64 years (fluoxetine), 60 years (amitriptyline)  Women: 44% (fluoxetine), 50% (amitriptyline) |
| Interventions | Fluoxetine 20-80 mg/d: N=9  Amitriptyline 100-300 mg/d: N=16 |
| Outcomes | HAMD, TESS |
| Informed consent | Not reported |
| Financial support | Not reported |
| CONSORT 2010 | 30% items fulfilled |

Yu XL 2004

| Methods | 4 week double blind RCT |
| --- | --- |
| Participants | Inclusion criteria:  Post-stroke Depression, Depressive episode according to CCMD-3  HAMD-24≥18, Age: /  Baseline Values:  HAMD score: 29.67+8.24 (citalopram), 31.02+8.82 (amitriptyline)  Age: 36-69 years, mean age 50 years  Women: 42% |
| Interventions | Citalopram 40 mg/d: N=30  Amitriptyline 150 mg/d: N=30 |
| Outcomes | HAMD, ADL, SSS, ASBS |
| Informed consent | reported |
| Financial support | Not reported |
| CONSORT 2010 | 24% items fulfilled |

Yuan YB 2011

| Methods | 8 week double blind RCT |
| --- | --- |
| Participants | Outpatients  Inclusion criteria:  Depressive episode according to DSM-IV, Age>18  HAMD-17≥15, CGI-S≥4, Age: /  Baseline Values:  HAMD score: 21.3 (duloxetine), 21.6 (paroxetine)  Mean age: 35 years (duloxetine), 33 years (paroxetine)  Women: 53% (duloxetine), 70% (paroxetine) |
| Interventions | Duloxetine 60 mg/d: N=121  Paroxetine 20 mg/d: N=123 |
| Outcomes | HAMD, HAMA, CGI, VAS |
| Informed consent | reported |
| Financial support | Not reported |
| CONSORT 2010 | 51% items fulfilled |

Zhang XL 2000

| Methods | 6 week double blind RCT |
| --- | --- |
| Participants | Inclusion criteria:  Depression according to CCMD-2-R  HAMD-17≥18, Age: /  Baseline Values:  HAMD score: 26.9+4.7 (paroxetine), 26.7+5.1 (amitriptyline)  Mean age: 40 years (paroxetine), 41 years (amitriptyline)  Women: 45% (paroxetine), 48% (amitriptyline) |
| Interventions | Paroxetine 20 mg/d: N=31  Amitriptyline 75 mg/d: N=31 |
| Outcomes | HAMD, CGI-SI, TESS |
| Informed consent | Not reported |
| Financial support | Not reported |
| CONSORT 2010 | 35% items fulfilled |

Zhang YL 2007

| Methods | 6 week double blind RCT |
| --- | --- |
| Participants | Inpatients  Inclusion criteria:  Depressive episode according to CCMD-3  HAMD-17≥18, Age: 20-60 years  Baseline Values:  HAMD score: 21.92+3.4 (citalopram), 28.96+6.9 (fluoxetine)  Mean age: 37 years (citalopram), 37 years (fluoxetine)  Women: 50% (citalopram), 53% (fluoxetine) |
| Interventions | Citalopram 10-40 mg/d: N=30  Fluoxetine 40-80 mg/d: N=30 |
| Outcomes | HAMD, TESS |
| Informed consent | Not reported |
| Financial support | Not reported |
| CONSORT 2010 | 32% items fulfilled |

Zhang Z 2001

| Methods | 6 week double blind RCT |
| --- | --- |
| Participants | Inclusion criteria:  Post-stroke Depression, Depressive episode according to CCMD-2-R  HAMD-17≥18, Age: /  Baseline Values:  HAMD score: 30.5+4.2 (sertraline), 31.4+4 (amitriptyline)  Mean age: 62 years  Women: 45% |
| Interventions | Sertraline 50-100 mg/d: N=31  Amitriptyline 75-225 mg/d: N=31 |
| Outcomes | HAMD, TESS |
| Informed consent | Not reported |
| Financial support | Not reported |
| CONSORT 2010 | 35% items fulfilled |

Zheng YS 2012

| Methods | 6 week double blind, double dummy RCT |
| --- | --- |
| Participants | In- and Outpatients  Inclusion criteria:  Depressive episode according to CCMD-3  Age: /  Baseline Values:  HAMD score: /  Age: 21-61 years (nefazodone), 20-60 years (fluoxetine)  Women: 34% (nefazodone), 36% (fluoxetine) |
| Interventions | Nefazodone: 300-400 mg/d: N=88  Fluoxetine: 20-40 mg/d: N=88 |
| Outcomes | HAMD, CGI |
| Informed consent | Not reported |
| Financial support | Not reported |
| CONSORT 2010 | 38% items fulfilled |

Zhou J 2005

| Methods | 6 week double blind RCT |
| --- | --- |
| Participants | Inpatients  Inclusion criteria:  Depression according to CCMD-3  HAMD-24>26, Age: /  Baseline Values:  HAMD score: 54.26+5.34 (paroxetine), 53.58+5.32 (imipramine)  Mean age: 35 years (paroxetine), 37 years (imipramine)  Women: 43% (paroxetine), 33% (imipramine) |
| Interventions | Paroxetine 20 mg/d: N=30  Imipramine 50-275 mg/d: N=30 |
| Outcomes | HAMD, HAMA, TESS |
| Informed consent | Not reported |
| Financial support | Not reported |
| CONSORT 2010 | 32% items fulfilled |

Zhou P 2012

| Methods | 8 week double blind RCT |
| --- | --- |
| Participants | Outpatients  Inclusion criteria:  Depression according to DSM-IV  HAMD-17≥20, Age: /  Baseline Values:  HAMD score: /  Mean age: 33 years (Duloxetine), 34 years (Paroxetine)  Women: 54% (Duloxetine), 83% (Paroxetine) |
| Interventions | Paroxetine: 20 mg/d, N=30  Imipramine: 50-275 mg/d, N=30 |
| Outcomes | HAMD |
| Informed consent | reported |
| Financial support | reported |
| CONSORT 2010 | 54% items fulfilled |

Zhu GK 2005

| Methods | 6 week double blind RCT |
| --- | --- |
| Participants | Inpatients  Inclusion criteria:  Depression according to CCMD  HAMD>18, Age: >65 years  Baseline Values:  HAMD score: 27.6+1.9 (citalopram), 26.7+1.7 (maprotiline)  Mean age: 69 years (citalopram), 70 (maprotiline)  Women: 40% (citalopram), 53% (maprotiline) |
| Interventions | Citalopram 20-40 mg/d: N=30  Maprotiline 25 mg/d: N=30 |
| Outcomes | HAMD, TESS |
| Informed consent | Not reported |
| Financial support | Not reported |
| CONSORT 2010 | 27% items fulfilled |

ADL: Activities of Daily Living

CCMD: Chinese classification of mental disorders

CGI: Clinical global impression

CONSORT: Consolidated Standards of Reporting Trial

DSM: Diagnostic and statistical manual of mental disorders

HAMA: Hamilton Anxiety Scale

HAMD: Hamilton Rating Scale of Depression

ICD: International classification of diseases

MADRS: Montgomery-Åsberg Depression Rating Scale

MAOIS: Monoamine Oxidase Inhibitor

MESSS: Mangled Extremity Severity Score

MMSE: Mini-Mental State Examination

NDS: Neural Function Deficient Scale

RCT: Randomised controlled trials

SDS: Self-rating Depression Scale

SERS: Åsberg Rating Scale for Side Effect

SSS: Scandinavian Stroke Scale

TCM: Traditional Chinese medicine

TESS: Treatment Emergent Symptom Scale

Appendix 4: Risk of bias of included studies

Low risk of bias Unclear risk of bias High risk of bias
